# Crowder‐specific modulation of hepatitis C virus NS3/4A protease activity and local structural dynamics

**DOI:** 10.1002/pro.70810

**Published:** 2026-10-08

**Authors:** Małgorzata Lobka, Joanna Trylska

**Affiliations:** ^1^ University of Warsaw Centre of New Technologies Warsaw Poland

**Keywords:** enzyme kinetics, fluorescence spectroscopy, hepatitis C virus, macromolecular crowders, NS3/4A protease, protein stability

## Abstract

Macromolecular crowding modulates enzyme behavior in crowder‐ and protein‐specific ways, yet its impact on viral proteases, which are often key therapeutic targets, remains unclear. Here, we investigated the hepatitis C virus NS3/4A protease under increasing concentrations of polyethylene glycols (PEGs), ficoll, dextran, and lysozyme using fluorescence‐based assays to examine enzyme kinetics, local Trp environments, thermal stability, and rotational restriction of the fluorescent probe in the surrounding medium. PEGs reduced catalytic activity while leaving substrate binding either unaffected or slightly enhanced. These effects were accompanied by a moderate tryptophan fluorescence spectral narrowing, consistent with reduced heterogeneity in local conformational environments. In contrast, ficoll enhanced catalytic efficiency despite stronger fluorescence quenching, indicating local structural changes that favored catalysis. Dextran and lysozyme inhibited protease activity through distinct kinetic patterns, likely reflecting differences in their size, shape, and chemical properties. Thermal analysis revealed crowder‐specific local structural changes in NS3/4A without global unfolding up to 65°C, with differences in local stability and flexibility corroborating the observed kinetic effects. Changes in the rotational restriction of the fluorescent probe further indicated that generalized restriction of molecular motion alone could not explain the effects of crowding on NS3/4A. These findings demonstrate that macromolecular crowding modulates NS3/4A catalysis through crowder‐specific effects on local structure.

## 1 INTRODUCTION

Although approximately 85% of a typical eukaryotic cell is water (Model et al., 2021), the remaining volume is densely packed with macromolecules such as proteins, lipids, polysaccharides, and nucleic acids (Fulton, 1982). This crowding, present in both cytoplasm and membranes, limits molecular freedom through steric exclusion. The excluded‐volume effect favors compact conformations and entropically stabilizes associations by minimizing surface area (Minton, 1981; Zhou et al., 2008). In contrast, the chemical nature of macromolecules gives rise to nonspecific interactions that can favor extended conformations by maximizing contact area (Rivas & Minton, 2016). Together, steric and soft interactions modulate the thermodynamics and kinetics of diffusion, folding, ligand binding, and stability (Alfano et al., 2024; Rivas & Minton, 2016; Zhou et al., 2008). These effects are particularly relevant for enzymes, whose activity depends on the structural flexibility required for substrate binding and catalysis (Agarwal, 2019; Henzler‐Wildman & Kern, 2007; Singh et al., 2024). For viral enzymes, folding determines not only catalytic competence but also engagement with host factors regulating replication and immune defense (Rampersad & Tennant, 2018). Understanding how the environment modulates viral enzyme function may therefore provide mechanistic insights and support therapeutic development. However, predicting how folding and kinetics are altered under crowded conditions remains challenging, as crowder size, shape, and chemical nature—together with enzyme‐specific properties—can modulate or override excluded‐volume effects (Acosta et al., 2017; Das et al., 2024; Ostrowska et al., 2019; Rivas & Minton, 2016; Speer et al., 2022).

The hepatitis C virus (HCV), a major cause of chronic liver disease and hepatocellular carcinoma (Manns et al., 2017), encodes the NS3/4A protease, which cleaves the viral polyprotein to release mature proteins essential for replication (Kolykhalov et al., 1994). In vivo, full‐length NS3‐NS4A is a bifunctional, multidomain, membrane‐bound complex consisting of an N‐terminal serine protease domain (aa 1–180) and a C‐terminal RNA helicase/NTPase domain (aa 181–631) within the NS3 protein. NS3 forms a heterodimer with the 54‐amino‐acid NS4A polypeptide (Yao et al., 1999). The NS3 N‐terminal amphipathic ɑ‐helix (aa 12–23), together with the N‐terminal 21 residues of NS4A, binds the complex to the endoplasmic reticulum membrane, positioning the protease domain at its surface (Brass et al., 2008; Yan et al., 1998). The protease domain adopts a chymotrypsin‐like fold stabilized by zinc, with catalytic organization dependent on NS4A binding (see Figure 1a). Zinc removal disrupts the native fold and disorganizes the catalytic triad (Kim et al., 1996; Stempniak et al., 1997). This zinc ion is structural rather than catalytic and is coordinated by Cys97, Cys99, Cys145, and His149 distal to the catalytic triad. Zinc is required for correct folding of the protease and is retained in the folded enzyme. Conversely, NS4A binding stabilizes the heterodimer and organizes the triad, particularly by positioning His57 for proteolysis (Yan et al., 1998; Zhu & Briggs, 2011). In the absence of NS4A, catalytic residues remain mobile and only transiently hydrogen‐bonded (McCoy et al., 2001). Beyond replication, the NS3/4A protease also cleaves the innate immune adaptors Cardif and Trif (Rebsamen et al., 2008), contributing to hepatitis C pathogenesis. The dual role makes NS3/4A protease a validated therapeutic target. Three generations of substrate‐mimicking inhibitors have been approved by the US Food and Drug Administration (Liverton, 2019), and compounds targeting non‐catalytic regions have been identified as well (El‐Araby et al., 2020). However, resistance mutations limit long‐term efficacy (Abuelizz et al., 2020). Since intracellular conditions can modulate protease function, studying this enzyme under crowding may reveal features absent in dilute solutions. While methods for studying NS3/4A in vivo are under active development (Chuang et al., 2022; Wang et al., 2010; Yao et al., 2016), few studies have examined NS3/4A under conditions mimicking intracellular complexity, either in silico (Brass et al., 2008; Ostrowska et al., 2021, 2023) or in vitro (Hamill & Jean, 2005; Popielec et al., 2020). In vitro, a soluble complex consisting of the NS3 protease domain and the central hydrophobic activation segment of NS4A (aa 21–32) embedded in the protease core is sufficient to recapitulate catalytic activity (Love et al., 1998); this optimized construct is hereafter referred to as NS3/4A (Figure 1a,b).

**FIGURE 1:**
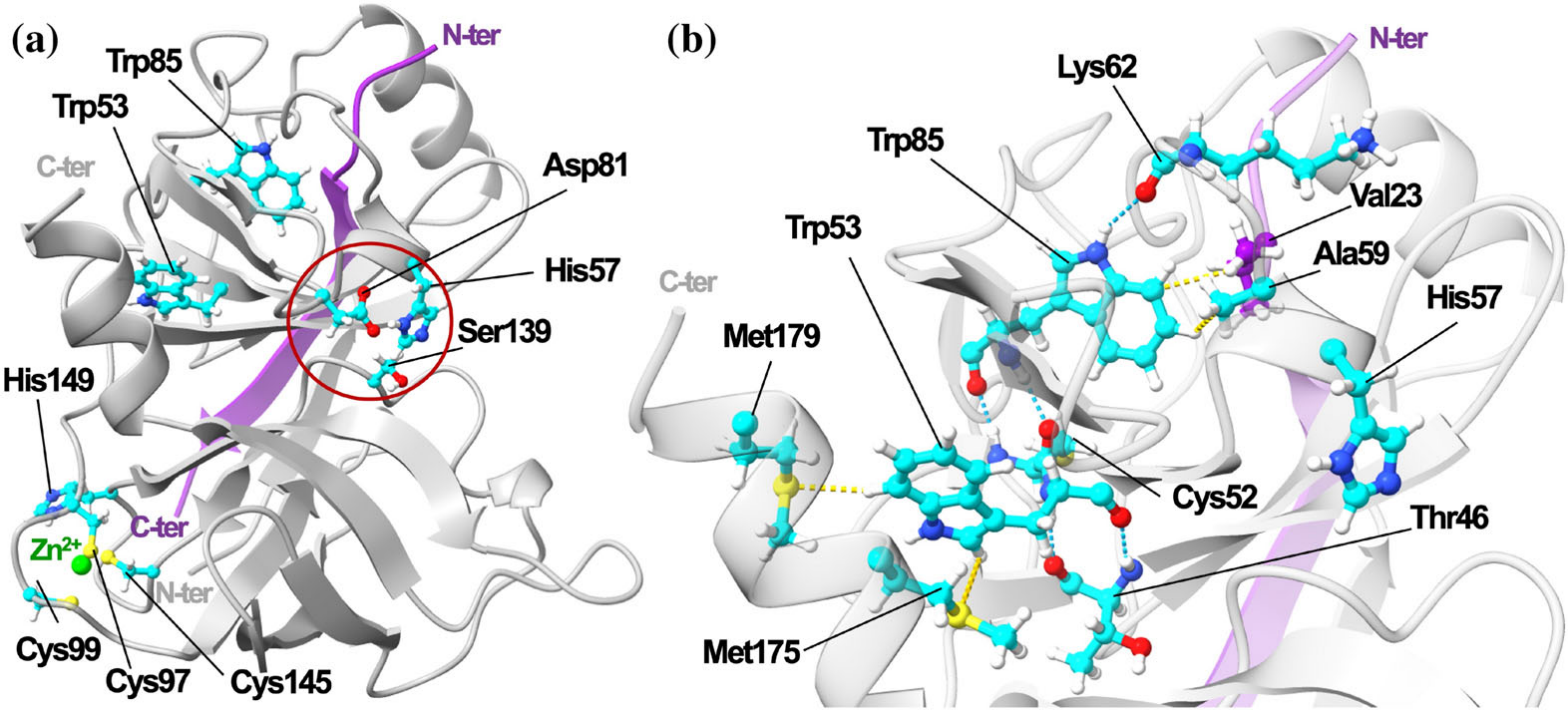
Structure of the NS3/4A protease functional complex. (a) Ribbon model of NS3 protease (gray) with NS4A (purple), visualized in ChimeraX (Meng et al., 2023), based on previous simulations (Popielec et al., 2020) and crystal structure Protein Data Bank ID: 4JMY (Lemke, 2013). Catalytic triad (His57, Asp81, and Ser139), zinc‐binding site (Cys97, Cys99, Cys145, His149; zinc ion in green), and tryptophan residues are highlighted in cyan. A red circle marks the active site. (b) Local environment of tryptophans with selected neighbors (for full contact lists, see Tables S2 and S3). Blue dashed lines indicate hydrogen bonds with distances of 2.8–3.1 Å between donor and acceptor; yellow dashed lines show selected hydrophobic contacts of 2.8–3.7 Å; Val23 (from NS4A) is shown in purple.

To mimic crowding in vitro, synthetic non‐ionic polymers such as polyethylene glycol (PEG), ficoll, and dextran are widely used. These polymers differ in their structure: PEG is a flexible linear polyether, whereas ficoll is a compact, highly branched sucrose‐based copolymer and dextran is a less branched, more coil‐like glucose polymer with a D‐glucose backbone (Biswas et al., 2018). Beyond simple steric exclusion, these agents can alter protein behavior through soft interactions. Interior PEG ether units ($-{\mathrm{CH}}_{2}{\mathrm{OCH}}_{2}-$; Figure S1A) form favorable hydrophobic contacts with aliphatic and aromatic carbons, while acting as hydrogen‐bond acceptors for backbone amides and cationic nitrogens. Because exposed nonpolar carbons dominate the protein surface during unfolding, these units tend to destabilize the native conformation. In contrast, PEG terminal hydroxyl groups ($-{\mathrm{CH}}_{2}\mathrm{OH}$) interact unfavorably with aliphatic carbons, but favorably with polar groups, contributing to protein stabilization when the aliphatic core remains buried (Knowles et al., 2015). Unlike PEG, ficoll and dextran interact primarily through their abundant hydroxyl groups (Figure S1A). These groups hydrogen bond with polar side chains and backbone carbonyl/amide groups, acting as both donors and acceptors. Moreover, they can restrict a water layer that bridges protein and carbohydrate surfaces (Redvanly et al., 2025). Recent all‐atom modeling of concentrated dextran solutions suggested how its hydroxyl‐rich, flexible chains organize the surrounding water layer and engage solute surfaces (Timr & Pluhařová, 2025). We have also observed in previous molecular dynamics (MD) simulations a water layer around tetra‐sucrose crowders mimicking ficoll (Ostrowska et al., 2023). Ultimately, these crowders shape protein behavior through a complex interplay of excluded volume, hydrogen bonding, hydrophobic contacts, electrostatic forces, and altered hydration environments (Rivas & Minton, 2016). PEG often reduces enzyme activity, as observed for oxidoreductases and hydrolases (Maximova et al., 2019; Sadarangani et al., 2023; Samanta et al., 2018), though activity enhancements have also been reported at low concentrations or with specific enzyme–substrate pairs (Wang et al., 2021). Ficoll more frequently enhances activity, as seen for dehydrogenases and transferases (Acosta et al., 2017; Li et al., 2019), though inhibition has been observed at high concentrations or in uncompetitive‐like systems (Matić et al., 2020; Rastogi & Chowdhury, 2021). Dextran most often reduces kinetic rates, especially above the chain‐overlap concentration (Homchaudhuri et al., 2006; Poggi & Slade, 2014; Rosario et al., 2025; Shahid et al., 2015), although increased activity at low concentrations has also been reported (Acosta et al., 2017). These crowders also affect protein folding. PEG can destabilize some proteins (Haas‐Neill et al., 2025; Muhammad et al., 2013), but in others it favors compact states (Sung et al., 2021). Ficoll generally stabilizes folding (Hong & Gierasch, 2010; Tokuriki et al., 2004), though destabilization of partially folded states has also been described (Kadumuri et al., 2016). Dextran tends to stabilize globular proteins (Cozzolino & Graziano, 2021; Shahid et al., 2015); however, its effect is not universally stabilizing and may even induce local destabilization or conformational changes (Singh & Chowdhury, 2013).

Given this variability, it remains unclear how crowding influences NS3/4A function. Our previous work showed opposing effects on catalytic activity and inhibition (Popielec et al., 2020): PEG 600, PEG 6000, and bovine serum albumin (BSA) reduced activity, whereas Ficoll 400 enhanced it, with the initial reaction rate changing in a concentration‐dependent manner. Ficoll also increased the inhibition constant for the antiviral drug telaprevir. Our previous MD simulations suggested that PEG slows substrate diffusion and increases substrate configurational entropy (Ostrowska et al., 2021), while ficoll‐mimicking crowders stabilize extended substrate conformations and promote productive substrate encounters near the NS3/4A catalytic triad (Ostrowska et al., 2023). Following these contrasting observations, we examined the effects of PEG and ficoll of different molecular weights, as well as Dextran 70 and lysozyme. The comparison of Ficoll 70 and Dextran 70 allowed us to examine polysaccharide crowders of similar nominal molecular weight but different chain organization, while lysozyme extended the analysis to a protein crowder with properties distinct from those of BSA. We determined kinetic parameters and combined these measurements with intrinsic Trp fluorescence and thermal stability assays to relate functional changes to the local protein environment. We also used the fluorescent molecular rotor DCVJ (9‐(2,2‐dicyanovinyl)julolidine) to assess rotational restriction in the surrounding medium. Together, these results allowed us to determine whether changes in NS3/4A activity follow a generalized restriction of molecular motion or arise from crowder‐specific effects.

## 2 RESULTS AND DISCUSSION

### 2.1 NS3/4A‐mediated substrate hydrolysis

The NS3/4A kinetics were analyzed under dilute and crowded conditions by determining initial rate ($v_{0}$), maximal rate ($v_{\max}$), Michaelis constant ($K_{M}$), turnover number ($k_{\mathrm{cat}}$), and catalytic efficiency ($k_{\mathrm{cat}}/K_{M}$). $K_{M}$ represents the substrate concentration at half‐maximal velocity and reflects both substrate affinity and turnover (Park, 2022). $k_{\mathrm{cat}}$ quantifies how many substrates are converted per enzyme per unit time and reflects the rate‐limiting step along the reaction pathway. The ratio $k_{\mathrm{cat}}/K_{M}$ describes catalytic performance under low substrate concentrations ($\left[S\right]\ll K_{M}$) (Park, 2022). Substrate‐concentration‐dependent changes in $v_{0}$ are shown in Figure 2a, and the derived kinetic parameters are summarized in Table 1 and Figure 3.

**FIGURE 2:**
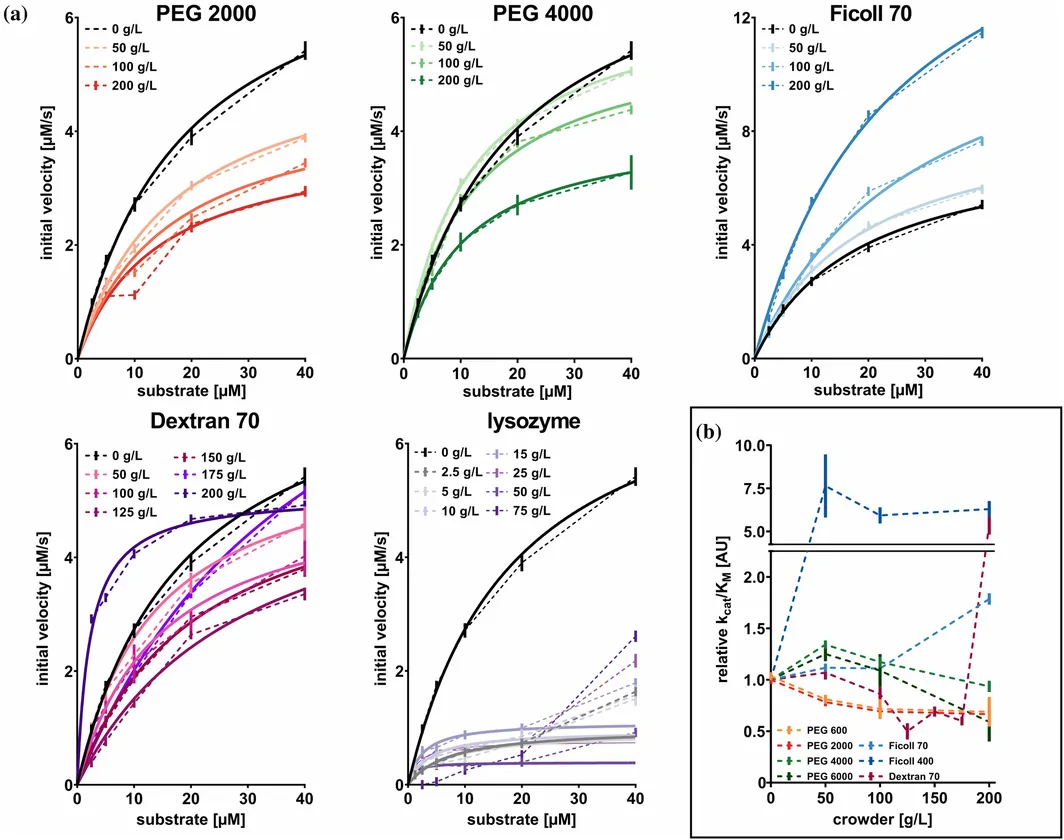
NS3/4A‐mediated hydrolysis kinetics under dilute and crowded conditions. (a) In this study, initial velocities were determined using 0–40 μM substrate and 0.05 μg/mL enzyme in dilute conditions, 50–200 g/L polymer crowders, and 2.5–75 g/L lysozyme. Data represent mean ± standard error of the mean (SEM) (*n* =3, each in duplicate) and were fitted to the Michaelis–Menten equation at the 0–40 μM range for polymer crowders and 0–20 μM for lysozyme (solid lines). Statistical significance of $v_{0}$ at 40 μM substrate relative to dilute control was assessed by two‐way analysis of variance (ANOVA) followed by Dunnett's post hoc test and was significant for all crowding agents and concentrations (*p* < 0.0001). (b) Comparison of calculated relative catalytic efficiencies ($k_{\mathrm{cat}}/K_{M}$), shown as mean ± SEM, with SEM obtained by standard error propagation, including the $K_{M}$ and $k_{\mathrm{cat}}$ covariance. Data for PEG 2000, PEG 4000, Ficoll 70, Dextran 70, and lysozyme were experimentally determined in this study. Data for PEG 600, PEG 6000, and Ficoll 400 were taken from (Popielec et al., 2020) and are shown for direct comparison.

**TABLE 1:** Kinetic parameters of NS3/4A in dilute and crowded conditions.

Crowder (g/L)		ϕ	${\left[S\right]}_{\mathrm{eff}}/\left[S\right]$	$v_{\max}$ (μM $s^{-1}$)	$K_{M}$ (μM)	$k_{\mathrm{cat}}$ ($s^{-1}$)	$k_{\mathrm{cat}}/K_{M}$ $\left({\mathrm{\mu M}}^{-1}s^{-1}\right)$
–	0	n/a	n/a	7.8±0.1	19±1	3900±100	205±5
PEG 2000	50	0.1	1.2	5.9±0.1	17±1	2800±100	165±5
	100	0.3	1.4	4.8±0.3	16±2	2300±100	144±12
	200	0.5	2.1	4.3±0.4	14±2	2200±200	157±11
PEG 4000	50	0.2	1.2	6.5±0.1	12±1	3300±100	275±16
	100	0.3	1.5	5.8±0.2	12±1	2900±100	242±13
	200	0.6	2.8	4.1±0.2	11±1	2100±100	191±10
Ficoll 70	50	0.2	1.2	8.9±0.2	19±1	4400±100	232±7
	100	0.4	1.6	13.4±0.6	29±2	6700±300	231±6
	200	0.8	4.0	19.0±0.5	25±1	9500±300	380±5
Dextran 70	50	0.5	2.0	6.1±0.2	14±1	3000±100	214±9
	100	≈1.0	67.1	5.4±0.3	15±2	2700±100	180±18
	125	n/a	n/a	6.0±0.5	30±4	3000±200	100±10
	150	n/a	n/a	5.8±0.5	21±1	2900±100	138±5
	175	n/a	n/a	11.0±0.4	45±3	5500±200	122±3
	200	n/a	n/a	5.1±0.1	3±1	2600±100	867±263
Lysozyme	2.5	<0.1	1.0	1.0±0.1	7±1	500±100	71±4
	5	<0.1	1.0	0.9±0.1	7±2	500±100	83±12
	10	<0.1	1.0	1.0±0.1	4±1	500±100	125±20
	15	<0.1	1.0	1.0±0.1	3±1	600±100	200±42
	25	<0.1	1.0	0.8±0.1	2±1	400±100	200±63
	50	0.1	1.1	0.4±0.1	1±1	200±100	200±125

*Note*: $v_{\max}$, $K_{M}$, and $k_{\mathrm{cat}}$ determined in this study were derived from Michaelis–Menten fits (Figure 2a) and are reported as mean ± SEM (*n* = 3, each in duplicate). SEM of $k_{\mathrm{cat}}/K_{M}$ was obtained by standard error propagation, including the $K_{M}$ and $k_{\mathrm{cat}}$ covariance. For each condition, excluded volume fractions (ϕ) and effective substrate concentration factors (${\left[S\right]}_{\mathrm{eff}}/\left[S\right]$) were calculated. Statistical significance of $v_{\max}$, $K_{M}$, and $k_{\mathrm{cat}}$ relative to dilute conditions is shown in Figure 3. n/a, not applicable.

**FIGURE 3:**
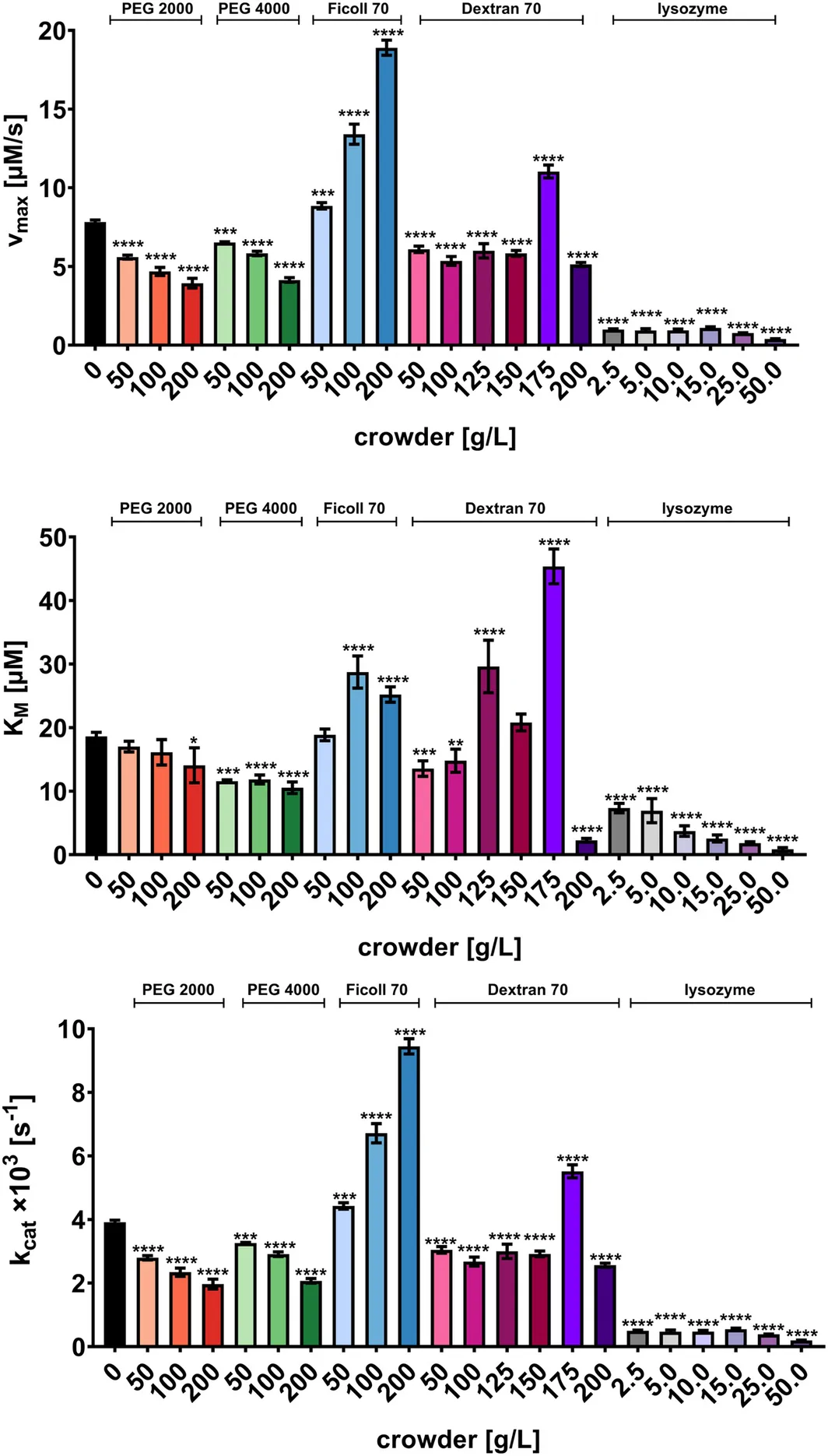
NS3/4A kinetic parameters in crowded conditions with their statistical significance relative to dilute control. $v_{\max}$, $K_{M}$, and $k_{\mathrm{cat}}$ determined in this study were derived from Michaelis–Menten fits (Figure 2a) and are reported as mean ±  SEM (*n* = 3, each in duplicate). Statistical significance of these parameters relative to dilute control was evaluated by two‐way ANOVA followed by Dunnett's post hoc test and is denoted as: ****p < 0.0001, ***p < 0.001, **p < 0.01, and *p < 0.05; otherwise, differences were not significant.

Under dilute conditions, NS3/4A catalytic efficiency at low substrate concentrations corroborated our previous report, but both $k_{\mathrm{cat}}$ and $K_{M}$ were approximately 2.3‐ to 2.7‐fold higher (Table S1). To investigate this discrepancy, we performed a side‐by‐side comparison of two enzyme‐handling protocols (Figure S2). In our previous work, the enzyme was thawed directly at room temperature (Popielec et al., 2020), whereas the revised protocol implemented here involved a preincubation on ice before room‐temperature equilibration to prevent thermal shock (Bhatnagar et al., 2005). Because all other experimental steps for the dilute control remained identical, this comparison demonstrates that the preincubation step altered NS3/4A kinetics, shifting its energy landscape such that the rate‐limiting transition state was unchanged but the energy of a preceding intermediate (e.g., the enzyme–substrate complex) was altered (Park, 2022). This effect is likely because preincubation on ice can promote local unfolding through ice recrystallization (Cao et al., 2003; Strambini & Gabellieri, 1996). Such a trade‐off (higher $k_{\mathrm{cat}}$ and $K_{M}$ but stable $k_{\mathrm{cat}}/K_{M}$) is consistent with cold‐induced conformational transitions (Saavedra et al., 2018).

The addition of PEG 2000 or PEG 4000 decreased both $k_{\mathrm{cat}}$ and $v_{\max}$ relative to dilute conditions (Table 1 and Figure 2a), reaching a 1.8‐ to 1.9‐fold reduction at 200 g/L PEGs. The NS3/4A catalytic efficiency remained relatively stable across PEG concentrations, consistent with our previous observations for PEGs with other masses (PEG 600 and PEG 6000, Figure 2b). Overall, all tested PEGs reduced turnover in a concentration‐dependent manner (Figure 3). Notably, $K_{M}$ decreased modestly in 200 g/L PEG 2000 and at all PEG 4000 concentrations. These results indicate that PEG does not hinder substrate binding but instead slows the following catalytic steps (Park, 2022). This hypothesis aligns with our previous MD simulations showing that PEG slows substrate diffusion but increases substrate flexibility, and that PEG alters the NS3/4A conformational dynamics (Ostrowska et al., 2023; Popielec et al., 2020). Together, these factors likely reduce the probability of catalytically competent alignment of the enzyme–substrate complex. The modest decrease in $K_{M}$ may partly reflect increased substrate availability due to excluded‐volume effects, with effective substrate concentrations rising up to 2.1‐ to 2.8‐fold in 200 g/L PEGs (Table 1). However, this increase did not compensate for the reduction in turnover, leading to decreased enzyme activity under saturating conditions. Similar PEG‐dependent inhibition, arising from competing excluded‐volume effects and soft interactions, has also been reported for other enzymes (Maximova et al., 2019; Sadarangani et al., 2023; Samanta et al., 2018).

In contrast, with Ficoll 70, the kinetic parameters gradually increased relative to dilute conditions, reaching up to a 2.4‐fold increase in reaction rate and 2.7‐fold increase in turnover at 200 g/L (Figure 2a and Figure 3). At 100–200 g/L of Ficoll 70, $K_{M}$ also increased, and at 200 g/L, the catalytic efficiency at sub‐saturating conditions improved by 1.8‐fold. A similar ficoll‐induced enhancement of $k_{\mathrm{cat}}$ and $k_{\mathrm{cat}}/K_{M}$ was previously observed with Ficoll 400 (Figure 2b). The increase in both $k_{\mathrm{cat}}$ and $k_{\mathrm{cat}}/K_{M}$ is consistent with lowering of the rate‐limiting transition‐state energy barrier (Park, 2022). This effect can arise from destabilization of the enzyme–substrate complex before the chemical reaction, stabilization of a catalytically competent configuration, or narrowing of the energy barrier driven by fast molecular fluctuations (Hay et al., 2010). Although these possibilities cannot be resolved with this kinetic assay, our previous MD simulations of NS3/4A surrounded by tetra‐sucrose molecules mimicking ficoll showed that this polymer forms soft interactions with the enzyme surface, stabilizes extended substrate conformations, and promotes productive encounters near the catalytic triad of NS3/4A (Ostrowska et al., 2023). This is consistent with the stronger stimulation of catalytic processing observed experimentally in this study. Despite improved turnover and catalytic efficiency, $K_{M}$ increased at 100–200 g/L Ficoll 70, indicating that the enzyme required a higher substrate concentration to reach $1/2v_{\max}$. However, the hydrophilic Ficoll 70 occupies up to 75% of the solution volume at 200 g/L, corresponding to nearly a four‐fold increase in effective substrate concentration (Table 1). Thus, the modest increase in $K_{M}$ likely reflects the competing excluded‐volume effects and soft interactions between ficoll, NS3/4A, and the Förster resonance energy transfer (FRET)‐substrate. Although Ficoll 400, studied previously, appeared more effective than Ficoll 70 (Figure S3), slight differences in the applied protocols preclude a direct comparison. Similar ficoll‐induced activity enhancement has also been reported for other enzymes (Acosta et al., 2017; Li et al., 2019; Rastogi & Chowdhury, 2021).

In 50–125 g/L Dextran 70, $v_{\max}$ and $k_{\mathrm{cat}}$ remained approximately 1.3‐ to 1.4‐fold lower than in dilute conditions (Table 1). The apparent $K_{M}$ decreased slightly at 50–100 g/L but subsequently increased up to 1.5‐fold at 125 g/L. Consequently, catalytic efficiency remained close to the dilute control value but then decreased to approximately half of that value at 125 g/L. Interestingly, at 150 g/L reaction rate and turnover remained similar to those at lower Dextran 70 concentrations, whereas the apparent $K_{M}$ moved back toward the dilute control value. Catalytic efficiency therefore partially recovered. At 200 g/L, turnover remained reduced and $K_{M}$ further decreased six‐fold relative to dilute conditions, resulting in an over fourfold increase in the apparent catalytic efficiency (Table 1, Figures 2b, 3, and S3). The effect at 175 g/L deviated from the observed trend, and we were not able to identify a definite cause. We therefore report these data in Figures 2 and S3, Table 1 for completeness but refrain from mechanistic interpretation. Overall, a consistent inhibitory effect was observed up to 150 g/L, followed by a pronounced change in the enzyme response at 200 g/L. This change occurred above the reported chain‐overlap concentration of Dextran 70, which is approximately 125 g/L in water. Above this threshold, individual polymer coils begin to interpenetrate and the solution enters a more complex regime (Xu et al., 2023). This can lead to pronounced kinetic alterations, as reported previously (Wilcox et al., 2020; Xu et al., 2023). In our case, the concentration dependence of the apparent $K_{M}$ began to change between 125 and 150 g/L, whereas the most pronounced decrease occurred at 200 g/L. The higher concentration required for a stronger effect may reflect differences between water and the assay buffer. Moreover, MD simulations of dextran hydrogels pointed to more pronounced structural and dynamical changes at concentrations exceeding chain overlap (Chen et al., 2013).

In contrast, Ficoll 70 has a higher overlap concentration of 250 g/L (Acosta et al., 2017), consistent with the absence of such a pronounced transition in its kinetic effects at 50–200 g/L. The spherical approximation used for excluded‐volume calculations also becomes invalid above 100 g/L Dextran 70 (Table 1), as the assumption of non‐overlapping hard‐sphere crowders no longer holds. Therefore, dextran interpenetration or further reorganization of its network may contribute to the shift in the kinetic trend observed here above 150 g/L. Dextran‐induced reduction in enzyme activity has been reported for other systems (Homchaudhuri et al., 2006; Poggi & Slade, 2014; Rosario et al., 2025). However, the opposite effects of ficoll and dextran on NS3/4A at 50–100 g/L are notable, as contrasting responses to polysaccharide crowders are relatively uncommon. Differences in magnitude have been reported previously (Homchaudhuri et al., 2006; Nasreen et al., 2021), whereas differences in the direction of kinetic changes have been described only in isolated cases, such as for yeast alcohol dehydrogenase (Wilcox et al., 2021). These behaviors may arise from differences in polymer shape, flexibility, and viscosity effects, as well as from soft interactions with proteins. For example, the stronger inhibition of alkaline phosphatase activity by dextran relative to ficoll has been attributed to dextran's extended, flexible structure, which introduces more physical barriers to enzyme–substrate encounters beyond viscosity effects alone (Homchaudhuri et al., 2006).

As a protein crowder, we previously used BSA and determined the NS3/4A activity with up to 7.5 g/L of BSA (Popielec et al., 2020). Here, we used hen egg‐white lysozyme (HEWL) as an alternative protein crowder. HEWL is small and compact (14 kDa, 129 amino acids), and is classified as a “hard” protein, in contrast to the larger, multidomain (66 kDa, 583 amino acids) and conformationally more adaptable (“soft”) BSA (Efimova et al., 2007). The two crowders also carry opposite net charges under our assay conditions (pH 7.8): BSA is anionic (pI ≈4.7; Peters, 1996), whereas HEWL is strongly cationic (pI ≈11.4; Wetter & Deutsch, 1951). We confirmed that the FRET‐substrate is not cleaved by HEWL at its highest concentration tested (Figure S4), which allowed monitoring NS3/4A activity up to 75 g/L of lysozyme. However, at the highest HEWL concentrations, the NS3/4A kinetics deviated from the Michaelis–Menten model, with successful fitting only up to 20 μM FRET‐substrate at 2.5–50 g/L of lysozyme (Figure 2a). The reaction rate continued to increase at 40 μM substrate without reaching a plateau, indicating non‐hyperbolic behavior. A similar deviation was observed at 75 g/L across all substrate concentrations. Such deviation at $\left[S\right]\gg K_{M}$ has been reported and may reflect allosteric effects or other regulatory phenomena (Baronas et al., 2021). This aligns with reports that lysozyme shows more pronounced effects on enzyme kinetics compared to polymer crowders or proteins with a weak crowding effect, such as BSA (Feig & Sugita, 2011; Wilcox et al., 2021). Within the concentration range where Michaelis–Menten model fitting was still valid, lysozyme reduced NS3/4A activity substantially. Already at 2.5 g/L, $v_{\max}$ and $k_{\mathrm{cat}}$ decreased approximately 7.5‐fold (Table 1). The concurrent 2.7‐fold decrease in $K_{M}$ partly compensated for the reduced turnover, resulting in an approximately 2.9‐fold decrease in catalytic efficiency. The kinetic parameters at 5–10 g/L were comparable to those at 2.5 g/L. Above 15 g/L, the reaction rate decreased further, with a nearly 20‐fold reduction in $v_{\max}$ and $k_{\mathrm{cat}}$ at 50 g/L relative to dilute conditions (Table 1). At this concentration, a corresponding 19‐fold reduction in $K_{M}$ restored the apparent catalytic efficiency to a value comparable to that in dilute conditions. This indicates that the rate‐limiting transition state was largely preserved (Park, 2022). Instead, lysozyme may have altered substrate binding dynamics or the enzyme's active conformation, leading to slower turnover despite higher apparent affinity. Its surface‐exposed lysines and arginines (Figure S1B) likely drive electrostatic and hydrogen‐bonding interactions (Ota & Takano, 2019) with both the NS3/4A enzyme and the anionic FRET‐substrate. NS3/4A has a highly asymmetric, positive electrostatic potential around the active site (Figure S5A), which may steer anionic peptide ligands, including our FRET‐substrate with a formal net charge of −5e at pH 7.8 (Figure S6A), into the catalytic region. Such steering most probably enhances the collision rate (Koch et al., 2001). Lysozyme's surface similarly creates a substantial electric field with extended positively charged regions (Figure S5B) that promotes collisions with anionic ligands (Ścibisz et al., 2017). At the ionic strength of our assay (100 mM NaCl; Debye length ≈1nm), long‐range electrostatic steering is largely screened, so these interactions would operate predominantly at short range, at or near contact (Israelachvili, 2011). We hypothesize that lysozyme acts as an electrostatic decoy, non‐specifically trapping the substrate in complexes with HEWL away from the protease. However, at elevated substrate concentrations (such as 40 μM tested here), this effect may be compensated by the remaining pool of free substrate available to drive the reaction. Nevertheless, this proposed mechanism remains a working hypothesis requiring validation in future studies. Such soft interactions are consistent with the modest excluded‐volume fraction (up to 10% at 50 g/L; Table 1), indicating that lysozyme effects arise primarily from direct interactions rather than from steric crowding alone. A lysozyme‐induced decrease in activity has also been reported for other enzymes (Okami et al., 2020; Poggi & Slade, 2014). To assess whether interactions between lysozyme and NS3/4A may contribute to the observed effects, we performed isothermal titration calorimetry (ITC) measurements by titrating concentrated lysozyme into an enzyme solution (see: Section 4.4). However, the low enzyme concentration limited the expected signal from interactions, while lysozyme produced concentration‐dependent heats of dilution, which were comparable to or larger than heat signals observed in the presence of NS3/4A (Figure S7). Together, this factors prevented reliable characterization of lysozyme‐NS3/4A interactions under crowded conditions.

In summary, these results show that PEG and ficoll consistently produce opposite effects on NS3/4A catalysis across various molecular weights: PEG reduces activity, while ficoll enhances it. Although also a polysaccharide, dextran inhibits NS3/4A activity but increases catalytic efficiency above its chain‐overlap concentration. Lysozyme exerts stronger inhibition than the synthetic polymers, even at lower concentrations. To assess whether these changes involve structural alterations, we further examined the local tryptophan environment of NS3/4A under polymer crowding. Lysozyme was excluded from the fluorescence analysis due to its intrinsic tryptophan fluorescence, which would obscure signal attribution to NS3/4A.

### 2.2 Crowder‐induced local structural changes near NS3/4A Trp residues

Protein dynamics are often dominated by minor conformational fluctuations, where the overall structural order remains intact but the distribution of accessible states changes (Kossiakoff, 1986). To probe such local changes in NS3/4A under crowded conditions, we monitored its intrinsic tryptophan fluorescence. At 35°C, close to the temperature used in the kinetic assay, fluorescence was detectable at protein concentrations as low as 1 μg/mL. Samples containing the protein in buffer and crowder‐containing solutions showed a significantly higher fluorescence yield than the background (Figure 4a,b), confirming a protein‐specific signal. In dilute conditions, the emission maximum was 315 nm (Figure 4a), consistent with tryptophans located in a predominantly hydrophobic environment (Rost & Sander, 1994) and supported by our analysis of the NS3/4A structure (Figure S8). Both Trp residues have negligible solvent exposure ($<5{Å}^{2}$). Trp53 is buried in the hydrophobic core between the NS3 β‐sheet and the C‐terminal ɑ‐helix (Figure 1a) and forms contacts with Val51, Leu82, Val83, and Tyr75 (2.2–3.5 Å; Table S2), and hydrogen bonds with Thr46 (2.9 Å; Figure 1b). The nearby Met175 and Met179 side chains (2.8–3.8 Å) may influence fluorescence through weak sulfur‐related quenching (Qiu et al., 2008). Trp53 also forms a small hydrophobic cavity positioned at the protein surface (Figure S8). Trp85 is positioned close to the NS3 surface loops and is stabilized by hydrogen bonds with Cys52 and Lys62 (2.8–3.1 Å; Table S3), and hydrophobic contacts with Leu64, Ile71, Val51, and Thr54 (1.8–3.4 Å). Although the reduced Cys52 is in proximity, it is not directly oriented toward the indole ring, suggesting low quenching probability (Qiu et al., 2008). Thus, both tryptophans report on tightly packed regions of NS3/4A and are well suited to detect local, rather than global, structural changes.

**FIGURE 4:**
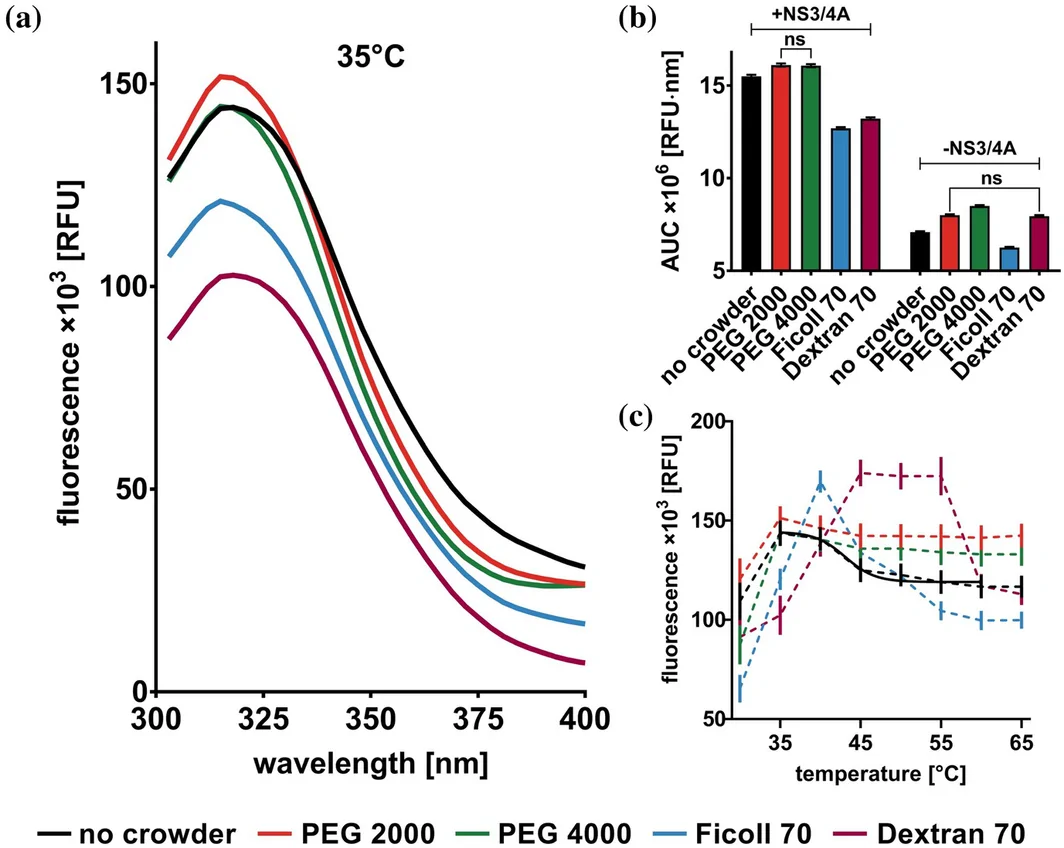
NS3/4A intrinsic fluorescence under dilute and crowded conditions. (a) Fluorescence emission spectra at 35°C were recorded with excitation at 280 nm for 1 μg/mL enzyme in dilute conditions, and in 200 g/L PEGs, 200 g/L Ficoll 70, and 150 g/L Dextran 70. Spectra were averaged from three scans, baseline‐corrected and smoothed with the Savitzky–Golay method. (b) Corresponding fluorescence yield with and without NS3/4A at 35°C quantified as the area under the emission curve (AUC; mean ± standard deviation [SD]). Statistical differences of AUC among all tested conditions were assessed by two‐way ANOVA followed by Tukey's post hoc multiple‐comparison test and were significant (*p* < 0.0001), unless stated otherwise (not significant, ns). Differences in AUC of samples with versus without NS3/4A within each condition were significant (*p* < 0.0001). (c) Baseline‐corrected intrinsic fluorescence intensity (mean ± SD; *n* = 2, three scans each) as a function of temperature. For dilute conditions, data were fitted to a Boltzmann sigmoidal curve across 35–60°C, with the midpoint estimated as 43.1°C. RFU, relative fluorescence units.

After adding 200 g/L of PEG 2000, the emission maximum red‐shifted by 3 nm, while the overall intensity remained similar relative to dilute conditions, indicating a slightly more polar Trp environment without major quenching (Figure 4a). In PEG 4000, the peak position did not change, but fluorescence yield decreased by 10% (Figure 4b). In both PEGs, the long‐wavelength tail contracted (Figure 4a), giving a narrower spectrum and suggesting reduced microenvironmental heterogeneity, often linked to restricted local flexibility (Kwok et al., 2021). Size‐dependent effects of PEG on fluorescence have been reported in other proteins (Fatima & Khan, 2007; Muhammad et al., 2013). Previous MD simulations showed broadly distributed PEG contacts with hydrophobic, aromatic, and sulfur‐containing residues of NS3/4A (Ostrowska et al., 2023), which may perturb local packing around Trp53 and Trp85. The subtle polarity increase in PEG 2000 likely reflects its shorter, more flexible chains accessing partially exposed hydrophobic pockets more effectively (Munasinghe et al., 2019). In contrast, the longer chains of PEG 4000 potentially limit penetration into these pockets, leading to different interaction patterns, for example, by displaying fewer ether oxygens for interactions (Begum & Matsuura, 1997). Since PEG also reduced NS3/4A activity, these fluorescence changes may reflect an altered structure near the catalytically relevant regions. For instance, Trp53 contacts both NS4A and the loop that positions His57 within the catalytic triad (Figure 1b). Even though in our previous MD simulations of NS3/4A surrounded by short 28‐mer PEG molecules, we did not observe PEG close contacts or interactions with the active site residues (Ostrowska et al., 2023; Popielec et al., 2020), PEG 4000 interactions with other NS3/4A residues may propagate to indirectly alter the dynamics of the active site.

In 200 g/L Ficoll 70, the NS3/4A fluorescence decreased by 20% in peak intensity and 30% in overall yield, while the emission maximum remained unchanged relative to dilute conditions (Figure 4a,b). The contraction of the long‐wavelength tail indicates a more uniform Trp microenvironment and reduced local heterogeneity (Kwok et al., 2021). However, inert crowding is typically expected to favor compaction and reduce solvent exposure of tryptophans, leading to increased fluorescence, as reported for the cellular retinoic acid‐binding protein (Hong & Gierasch, 2010). In contrast, the observed decrease in fluorescence intensity without a major change in polarity points to enhanced non‐radiative quenching, often by ligands or neighboring residues (Anand et al., 2011; Chen et al., 2006). Previous MD simulations indicate that ficoll interacts more extensively with NS3/4A than PEG, forming numerous hydrogen bonds with the protein surface (Ostrowska et al., 2023). These simulations also showed that ficoll‐mimicking crowders altered NS3/4A conformational flexibility to a greater extent than PEG. The branched polysucrose structure and flexibility of ficoll in ionic solution (Khan et al., 2024; Venturoli & Rippe, 2005) likely facilitate contacts with the protein, which may promote static quenching of Trp residues through proximity to Met residues or ficoll hydroxyls, as reported for fibrinogen (Sankaranarayanan & Meenakshisundaram, 2016). Despite reduced fluorescence, the NS3/4A activity increased in the presence of ficoll, indicating that the protease remained functionally folded (Stempniak et al., 1997). These findings suggest that ficoll may perturb local environments without inducing global unfolding, consistent with the studies on the α‐subunit of tryptophan synthase, where Ficoll 70 induced local loosening while the overall fold remained intact (Kadumuri et al., 2016). Fluorescence changes near Trp53, which lies at the interface critical for NS4A binding and catalytic alignment, point to localized rearrangements that coincide with increased enzymatic efficiency in ficoll.

In 150 g/L Dextran 70, NS3/4A fluorescence was strongly reduced: the maximum intensity decreased by 40%, the overall fluorescence yield dropped by 60%, and the emission maximum red‐shifted by 3 nm relative to dilute conditions (Figure 4a,b). The long‐wavelength tail contracted more than in any other crowder, indicating strongly reduced microenvironmental heterogeneity around Trp residues (Kwok et al., 2021). The combination of marked intensity loss with only a modest red‐shift suggests that Trp53 and Trp85 become slightly more solvent‐exposed while also experiencing enhanced non‐radiative quenching. This subtle difference in local perturbation between the two polysaccharide crowders is consistent with previous reports of divergent effects on proteins such as apo‐myoglobin (Nasreen et al., 2021), and with the distinct influence of Dextran 70 versus Ficoll 70 on NS3/4A kinetics.

These results reveal distinct local structural perturbations in NS3/4A under different crowding conditions. PEGs caused modest polarity shifts and reduced spectral heterogeneity, consistent with restricted local flexibility. In contrast, Ficoll 70 and Dextran 70 produced stronger fluorescence quenching; results with ficoll suggested enhanced non‐radiative interactions, while those with dextran indicated greater polarity changes, both pointing to crowder‐specific rearrangements near buried Trp residues without global unfolding.

### 2.3 NS3/4A conformational response near Trp residues to moderate heating under crowding

We further assessed local thermal unfolding profiles using temperature‐dependent fluorescence. Increasing temperature from 30 to 65°C progressively altered the fluorescence spectra under all conditions (Figure S9A), indicating structural changes around the Trp residues. Across this range, the 350/330 nm fluorescence ratio remained below the threshold at which cooperative unfolding transitions can be reliably detected (<0.67 (Žoldák et al., 2017); Figure S9B), and changes in the ratio were not statistically significant.

From 30 to 35°C, fluorescence intensity increased by more than 25% in buffer, PEGs, and Ficoll 70 (Figure 4c). The emission maximum blue‐shifted by 6–9 nm (Figure S9A), suggesting a more homogeneous and less polar local environment, consistent with tightening or ordering around Trp residues (Kwok et al., 2021). In Dextran 70, this initial intensity increase was absent, indicating no additional stabilization upon mild heating. In buffer, fluorescence remained stable up to 40°C. Above this temperature, intensity decreased by more than 10% and the emission maximum red‐shifted to 324 nm, indicating progressive local solvent exposure (Rost & Sander, 1994). A midpoint of approximately 43°C was estimated for these local changes (Figure 4c). Notably, no abrupt transition was observed up to 65°C, suggesting that global fold stability is preserved throughout this range, consistent with the buffer formulation designed to stabilize NS3/4A for in vitro assays (Urbani et al., 1999). As mild heating has been reported to trigger hydrophobicity‐driven local folding primarily in natively disordered proteins (Uversky, 2013), these observations align with our earlier hypothesis of an incompletely folded conformation after sample thawing on ice.

In 200 g/L PEG 2000 and PEG 4000, fluorescence intensity increased slightly during the initial heating step and then remained stable up to 65°C (Figure 4c), indicating no major temperature‐induced perturbations in the local Trp environment. The emission maximum stayed between 315 and 318 nm across the entire temperature range (Figure S9A), consistent with a largely unchanged local polarity around both Trp residues (Kwok et al., 2021). Although the overall temperature profiles were similar for the two PEGs, fluorescence yield was slightly higher in PEG 2000 than in PEG 4000, suggesting subtle molecular‐weight‐dependent differences in how PEG interacts with the protein. PEG has previously been reported to stabilize local structural elements during heating (Lencastre Novaes et al., 2010; Rawat et al., 2010; Rodríguez‐Martínez et al., 2008; Wang et al., 2021). Consistently, PEG has been shown to reshape the conformation and dynamics of *Escherichia coli* prolyl‐tRNA synthetase through combined crowding and confinement effects (Liebau et al., 2024). Together with the kinetic data, these fluorescence and thermal profiles indicate that PEG does not unfold NS3/4A, but likely restricts local flexibility. This reduction in conformational sampling may underlie the observed decrease in catalytic efficiency. These observations offer a potential structural explanation for why PEG slows catalytic steps while leaving substrate binding largely intact.

In 200 g/L Ficoll 70, fluorescence intensity increased up to 40°C and the emission maximum blue‐shifted to 312 nm (Figures 4c and S9A), indicating a more hydrophobic Trp environment and reduced collisional quenching (Kwok et al., 2021). This suggests that local packing around Trp residues becomes tighter upon moderate heating. Above 40°C, however, fluorescence intensity dropped sharply and the emission maximum shifted progressively to 324 nm by 50°C, reflecting increased polarity and local unfolding (Kwok et al., 2021). Beyond this point, the signal declined only slightly up to 65°C. Together, these patterns indicate that ficoll does not stabilize local structure during modest heating, but instead alters local folding in a more complex way than simply shifting the local unfolding midpoint. In fact, a complex influence of ficoll on protein folding was previously reported, for example, for the recombinant Rab2B protein, where Ficoll promoted the formation of partially unfolded states distinct from both native and fully unfolded conformations (Nath et al., 2025). This ficoll‐specific pattern contrasts with the behavior of dextran, whose linear, flexible chains engage the protein differently and produce distinct structural and kinetic consequences.

In 150 g/L Dextran 70, fluorescence intensity increased markedly between 35 and 45°C, remained stable up to 55°C, and then decreased sharply at 60°C (Figure 4c). The emission maximum stayed at 315 nm through 55°C and shifted to 324 nm at 60°C (Figure S9A), indicating a transition toward a more polar Trp environment (Kwok et al., 2021). This pattern suggests an initial reduction in local flexibility around tryptophans, followed by a plateau and then local unfolding at higher temperatures. Both polysaccharide crowders promoted a less polar, more shielded Trp environment during early heating, but Dextran 70 additionally maintained this locally folded state over the 45–55°C range. Dextran‐induced thermal stability has been reported for other proteins (Mikaelsson et al., 2014; Sasahara et al., 2003). This extended local stability aligns with the reduced turnover observed in dextran, suggesting that stabilization of non‐productive conformations may limit catalytic efficiency.

These results reveal that NS3/4A undergoes local structural changes without global unfolding up to 65°C. PEGs preserved spectral stability, consistent with NS3/4A restricted flexibility, while ficoll and dextran showed distinct thermal responses: Ficoll 70 triggered early destabilization, whereas Dextran 70 increased local structural integrity at lower temperatures and maintained it up to 55°C. These crowder‐specific effects parallel their kinetic profiles, suggesting that local stability and flexibility modulate catalytic efficiency under crowded conditions.

### 2.4 Crowder‐dependent changes in DCVJ rotational freedom

To distinguish changes intrinsic to NS3/4A from general effects of the medium on molecular mobility, we used the molecular rotor DCVJ, whose intramolecular rotation provides a non‐radiative decay channel (Figure S10A), so that restricting this motion increases the fluorescence quantum yield (Gutkowski et al., 2006). Because the DCVJ signal also depends on temperature, solvent polarity, and specific interactions with its environment (Haidekker & Theodorakis, 2010), we interpret it as reporting rotational restriction rather than bulk or local viscosity.

At 35°C, 4 μM DCVJ fluoresced significantly above the corresponding background in all conditions, including 200 g/L Ficoll 70, which had a high intrinsic signal (Figure 5a,b). Yields in water and assay buffer were comparable, supporting the use of the buffer as the dilute reference. The broader spectrum in water (Figure 5a) probably reflects heterogeneous dye solvation (Haidekker et al., 2005). In 200 g/L PEGs, the fluorescence rose by approximately 18%, independently of PEG molecular weight (Figure 5b), indicating partial restriction of DCVJ rotation (Ehrlich et al., 2024). This agrees with our previous MD simulations, in which PEG slows substrate diffusion and alters the dynamics of both substrate and enzyme (Ostrowska et al., 2023; Popielec et al., 2020). Because PEG reduced turnover rather than apparent substrate binding, moderate restriction of mobility appears to accompany changes in post‐binding conformational dynamics likely driven by PEG‐specific interactions.

**FIGURE 5:**
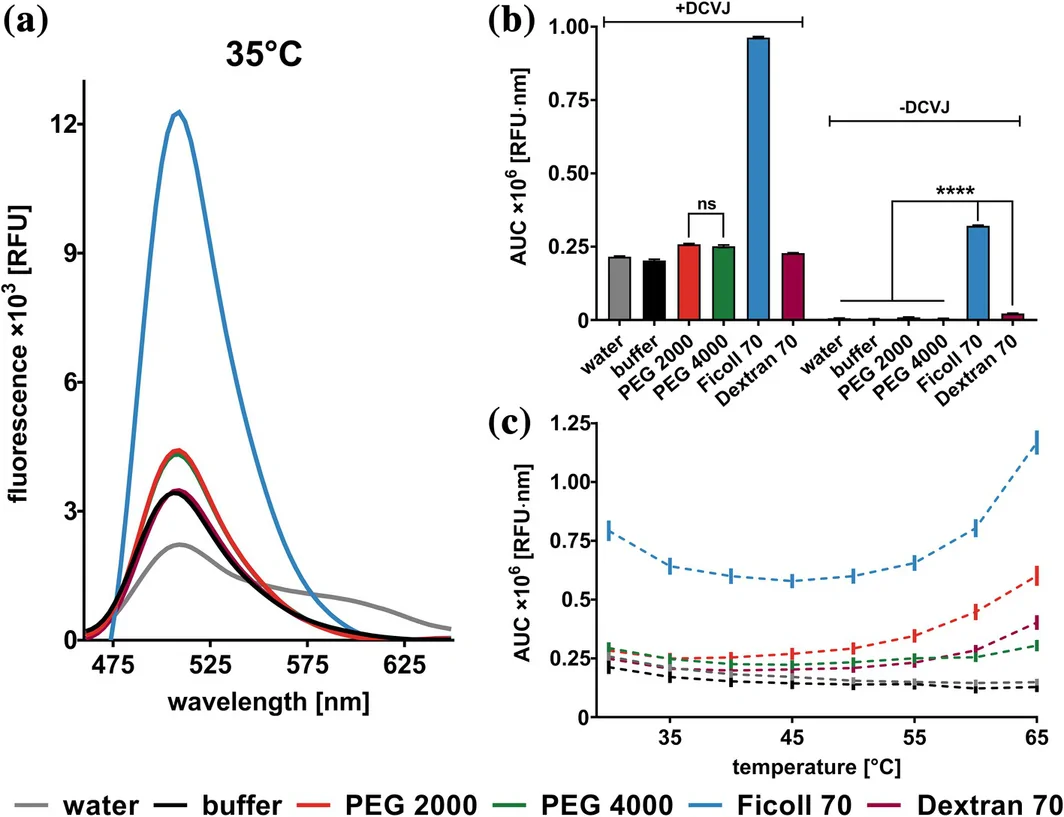
DCVJ fluorescence under dilute and crowded conditions. (a) Fluorescence emission spectra at 35°C were recorded with excitation at 440 nm for 4 μM DCVJ in dilute conditions (water and buffer), and in 200 g/L PEGs, 200 g/L Ficoll 70, and 150 g/L Dextran 70. Spectra were averaged from three scans, baseline‐corrected and smoothed with the Savitzky–Golay method. (b) Corresponding fluorescence yield with and without DCVJ at 35°C quantified as AUC (mean ± SD). Statistical differences in AUC among all tested conditions were assessed by two‐way ANOVA followed by Tukey's post hoc multiple‐comparison test and considered significant for *p* < 0.05. For clarity, only selected comparisons used in the discussion are shown: DCVJ in PEG 2000 versus PEG 4000 and DCVJ in water versus buffer were not significant (ns), whereas Ficoll 70 background differed significantly from the other backgrounds (****p < 0.0001). Differences in AUC of samples with versus without DCVJ within each condition were significant (*p* < 0.0001). (c) Baseline‐corrected DCVJ fluorescence yield quantified as AUC (mean ± SEM; *n* = 3, three scans each) as a function of temperature.

Ficoll 70 (200 g/L) produced the strongest response, a 3.2‐fold increase in DCVJ fluorescence yield (Figure 5a,b), consistent with substantial rotational restriction (Ehrlich et al., 2024), although specific dye‐crowder interactions may also contribute. Yet ficoll also increased catalytic activity, so restricting a small probe did not suppress catalytically relevant dynamics. Conversely, 150 g/L Dextran 70 gave a yield indistinguishable from buffer (Figure 5a,b), even though this concentration exceeds the chain‐overlap concentration (Xu et al., 2023), while NS3/4A intrinsic fluorescence and activity both changed markedly. Dextran creates heterogeneous local environments that hinder only some probe subpopulations (Goins et al., 2008); DCVJ may therefore remain mobile in solvent‐rich pockets between interpenetrating chains, whereas the larger enzyme encounters the meshwork. These opposing polysaccharide effects cannot be explained by uniform restriction of mobility or by bulk viscosity, and instead support a role for crowder‐specific interactions, consistent with the extensive contacts between ficoll‐mimicking crowders and the NS3/4A surface seen in previous simulations (Ostrowska et al., 2023).

Temperature profiles were non‐monotonic and condition‐dependent (Figures 5c and S10B). Fluorescence first fell between 30 and 40–45°C in all samples, consistent with greater rotational freedom as viscosity decreases and with the intrinsic temperature dependence of the probe (Howell et al., 2012). Above this range it remained stable in water and buffer but rose in every crowder solution, which viscosity alone cannot explain (Howell et al., 2012) and which more likely reflects altered dye solvation, partitioning, or crowder interactions (Aniruddha & Anunay, 2008; Haidekker et al., 2005). These profiles did not track the intrinsic fluorescence of NS3/4A. In PEGs, particularly PEG 2000, the DCVJ signal rose while the protein signal remained comparatively stable. In Ficoll 70, DCVJ rose above approximately 45°C whereas NS3/4A fluorescence peaked near 40°C and then decreased. In Dextran 70, DCVJ rose progressively above 45°C while NS3/4A fluorescence was stable to 55°C before dropping sharply (Figure 4c). DCVJ and the Trp environments therefore report on different features of the local environment during heating.

Overall, the tested crowders generate distinct local physicochemical environments. The absence of a simple relationship between DCVJ fluorescence, NS3/4A intrinsic fluorescence, and catalytic activity indicates that generalized restriction of molecular motion alone does not determine the protease response. Crowder‐specific interactions and selective modulation of local protein dynamics are the more likely origin.

### 2.5 Scope and limitations of the crowding model

In this study we used a simplified model to investigate how macromolecular crowding alters NS3/4A activity and local structure. By combining the minimal catalytically competent NS3/4A construct with defined synthetic and protein crowders, we bridged the gap between standard dilute assays and the complex intracellular environment, capturing two key features of the cellular interior: physical volume exclusion and crowder‐specific chemical interactions.

Several limitations follow from this design. First, the construct lacks the full‐length, multidomain organization of the NS3/4A complex. Second, the enzyme is evaluated in bulk solution rather than anchored to the host endoplasmic reticulum membrane. Third, we used isolated, homogeneous crowders rather than the heterogeneous, multi‐component cytoplasm of the host cell. Next, because we worked with a commercial NS3/4A construct, we could not perform site‐directed mutagenesis to generate a Trp‐free control or an engineered Trp FRET‐pair that would rule out direct crowder‐induced quenching. MD simulations with dextran‐like and lysozyme crowders would likewise be needed to capture conformational changes directly. Consequently, our model system maps foundational crowding physics but cannot predict quantitative behavior in a replicating virus. Any downstream clinical implications must be validated in membrane‐associated and live‐cell replication models.

## 3 CONCLUSIONS

We demonstrated that macromolecular crowding modulates HCV NS3/4A protease activity and thermal response in a crowder‐specific manner. PEG impaired catalysis by reducing turnover and altering local flexibility without unfolding the protein. In contrast, ficoll enhanced catalytic efficiency and promoted local structural changes favorable for activity. Dextran and lysozyme inhibited activity through distinct mechanisms, likely reflecting differences in polymer structure and protein interactions. These functional effects paralleled changes in tryptophan fluorescence and thermal stability, highlighting that local structural dynamics, rather than global unfolding, play a key role in how this viral protease responds to crowded environments. DCVJ measurements further showed that crowder‐dependent restriction of probe rotation did not directly correspond to NS3/4A fluorescence or catalytic activity, indicating that generalized restriction of molecular motion or bulk viscosity alone cannot explain the protease response. These results align with broader effects reported for other enzymes and reveal NS3/4A‐specific features that remain hidden in dilute conditions. Together, they indicate that the identity of the crowder, not merely the excluded volume it creates, determines how this viral protease behaves in a crowded environment.

## 4 MATERIALS AND METHODS

The HCV NS3/4A protease (genotype 1b, strain HC‐J4) (Taremi et al., 1998), polymer crowders, DCVJ, Fmoc (9‐fluorenylmethoxycarbonyl)‐protected amino acids, L‐(+)‐lactic acid, TentaGel S RAM resin (0.24 mmol/g loading), and standard peptide synthesis reagents were obtained from Sigma‐Aldrich. HEWL, dithiothreitol (DTT), and glycerol were from Carl Roth GmbH. EDANS (5‐((2‐aminoethyl)amino)naphthalene‐1‐sulfonic acid) was from AnaSpec Inc. All reagents were of analytical or reagent grade and were used without further purification. Data analysis and visualization were performed using GraphPad Prism v8.0.

### 4.1 FRET‐substrate synthesis and purification

The NS3/4A fluorogenic substrate Ac‐DED(EDANS)EEAbu‐ψ‐[COO]‐ASK(DABCYL)‐NH_2_ (Taliani et al., 1996) was used (Figure S6A). This substrate contains a FRET pair, in which the fluorophore EDANS is quenched by DABCYL (4‐((4‐(dimethylamino)phenyl)azo)benzoic acid). Enzymatic cleavage separates the EDANS fluorophore and DABCYL quencher, reducing FRET efficiency, and thereby restoring fluorescence. The FRET‐substrate was synthesized manually on a 120 μmol scale using the Fmoc‐based solid‐phase peptide synthesis (SPPS) on Rink amide resin, as previously described (Popielec et al., 2020). After cleavage and side‐chain deprotection in a trifluoroacetic acid (TFA)/water/triisopropylsilane solution (95:2.5:2.5 v/v/v, 2 h), the crude depsipeptide was purified by reversed‐phase high‐performance liquid chromatography (RP‐HPLC) on Knauer Eurospher II 100–5 C18 columns (250 × 4.6 mm analytical; 250 × 8 mm semipreparative). A binary solvent system (S 1132, Sykam GmbH) with a 35%–45% acetonitrile gradient in water containing 0.1% TFA was applied over 30 min at 1.5 mL/min (analytical) and 4.5 mL/min (semipreparative). The depsipeptide, monitored at 220 nm, eluted at 8.5 min (Figure S6B). The purified FRET‐substrate had a purity of >95% as determined by analytical HPLC. Identity was confirmed by electrospray ionization mass spectrometry (ESI‐MS; Q‐TOF Premier, Waters), yielding a mass‐to‐charge ratio of m/z=1548.6, consistent with the calculated value ${\left[M+H\right]}^{+}=1548.6$ (Figure S6C). TFA counterions were exchanged for chloride by dissolution in 100 mM HCl, followed by freezing and lyophilization.

### 4.2 Buffer and sample preparation

The assay buffer contained 50 mM 4‐(2‐hydroxyethyl)‐1‐piperazineethanesulfonic acid pH 7.8, 100 mM NaCl, 20% v/v glycerol and 5 mM DTT, and was used for all dilutions. NS3/4A was diluted as needed and stored at −80°C. FRET‐substrate (400 μM in assay buffer) and EDANS (1 mM in dimethyl sulfoxide [DMSO]) were diluted as needed and stored at −20°C. DTT‐containing solutions were thawed once and used immediately. Lysozyme was dissolved in assay buffer at room temperature, while polymer crowders were dissolved in assay buffer without DTT by mixing overnight at 35°C and 200 rpm. DTT was added fresh before enzyme incubation. NS3/4A was kept on ice, followed by 10 min preincubation at room temperature.

### 4.3 EDANS fluorescence measurement

Fluorescence was measured at 37°C in black 96‐well F‐bottom plates (100 μL/well; FLUOTRAC, Greiner Bio‐One) using a Synergy H1 Hybrid microplate reader (BioTek) in top‐read mode (7 mm read height, autogain enabled). Excitation and emission wavelengths were 340 and 490 nm, respectively. Plates were shaken at 37°C and 200 rpm for 30 s and allowed to settle for 30 s before the measurement. Samples without crowder (0 g/L) served as dilute controls.

#### 4.3.1 EDANS calibration

Calibration was performed in buffer and in crowder solutions using serial EDANS dilutions (0–1000 nM final). The final crowder concentrations were: 0, 50, 100, and 200 g/L for PEGs and Ficoll 70; 0, 50, 100, 125, 150, 175, and 200 g/L for Dextran 70; and 0, 15, 25, 50, and 75 g/L for lysozyme. EDANS and crowders were preincubated separately at 37°C for 10 min, then mixed in plates. Fluorescence was recorded in three independent runs (*n* = 3), each performed in duplicate. Fluorescence was baseline‐corrected, reported as mean ±SEM in RFU, and showed a linear response across all tested conditions ($R^{2}>0.9$; Figure S11A). Samples without EDANS (0 nM) served as background controls. To test for inner‐filter effects, serial FRET‐substrate dilutions (0–40 μM final) were prepared with and without 500 nM EDANS. Substrate and crowders were preincubated separately at 37°C for 10 min, then mixed in plates. Fluorescence was measured in two independent runs (*n* = 2), each performed in duplicate. Fluorescence was baseline‐corrected and reported as mean ± SEM in RFU. Samples without FRET‐substrate (0 μM) served as background controls. As the response remained linear ($R^{2}>0.9$; Figure S11B), no inner‐filter corrections were applied in the kinetic assay.

#### 4.3.2 Enzyme kinetics assay

Kinetic measurements were performed with NS3/4A (0.05 μg/mL final, [$E_{\mathrm{tot}}$]) in buffer and crowder solutions, using serial FRET‐substrate dilutions (0–40 μM final, [*S*]). Final crowder concentrations were: 0, 50, 100, and 200 g/L for PEGs and Ficoll 70; 0, 50, 100, 125, 150, 175, and 200 g/L for Dextran 70; and 0, 2.5, 5, 10, 15, 25, 50, and 75 g/L for lysozyme. Enzyme in buffer/crowder solution and substrate (in buffer only) were preincubated separately at 37°C for 10 min before mixing in the plate. Fluorescence was recorded every 45 s for 60 min in three independent runs (*n* = 3), each performed in duplicate, and baseline‐corrected. Samples without FRET‐substrate (0 μM) served as background controls. Additional measurements in the absence of NS3/4A were performed in two independent runs (*n* = 2) under dilute conditions and in the presence of lysozyme at the highest tested concentration (75 g/L) to verify that no spontaneous FRET‐substrate cleavage or HEWL‐mediated hydrolysis occurred. The fluorescence was baseline‐corrected as before and reported as mean ± SD in RFU (Figure S4). Initial reaction velocities ($v_{0}$) were determined from the linear phase of fluorescence increase and fitted to the Michaelis–Menten equation ($R^{2}>0.9$, except $R^{2}>0.8$ in 50 g/L lysozyme; Figure 2a): $v_{0}=v_{\max}\left[S\right]/\left(K_{M}+\left[S\right]\right)$, where $v_{\max}$ is maximal reaction rate, $K_{M}$ is the Michaelis constant, and catalytic turnover is described as $k_{\mathrm{cat}}=v_{\max}/\left[E_{\mathrm{tot}}\right]$. The statistical analysis of $v_{0}$ at 40 μM FRET‐substrate and derived kinetic parameters, reported as mean ± SEM, was performed using a two‐way ANOVA with crowder type and concentration as independent variables, followed by Dunnett's post hoc test to compare each crowding condition with the dilute control. Multiplicity‐adjusted *p* values <0.05 were considered statistically significant. SEM of $k_{\mathrm{cat}}/K_{M}$ was propagated from derived $k_{\mathrm{cat}}$ and $K_{M}$ values using standard error propagation, including their covariance. The excluded‐volume fraction was estimated by approximating crowders as spheres with reported hydrodynamic radii: 1.27 nm and 1.72 nm for PEG 2000 and PEG 4000 (Lee et al., 2009), respectively, 4.70 nm for Ficoll 70 (Georgalis et al., 2012), 6.49 nm for Dextran 70 (Armstrong et al., 2004), and 1.90 nm for lysozyme (Grisham & Nanda, 2018). The excluded volume fraction was calculated as: $\phi =\left[C\right]N_{A}\left(4/3\right)\pi r_{h}^{3}/M$, where [*C*] is the crowder w/v concentration, $N_{A}$ is Avogadro's number, and *M* is the crowder molecular weight (Li et al., 2020). The substrate concentration corrected for excluded‐volume effects was calculated as: ${\left[S\right]}_{\mathrm{eff}}=\left[S\right]/\left(1-\phi \right)$.

### 4.4 Isothermal titration calorimetry of lysozyme and NS3/4A

The ITC assay was performed using a power‐compensated Nano ITC (TA Instruments) equipped with a 190 μL gold cell (165 μL working volume) and a 50 μL syringe. Lysozyme was dissolved in the assay buffer. Buffer and lysozyme solutions were degassed with gentle mixing for 30 min at room temperature, while NS3/4A was degassed for 10 min. Samples were brought to 37°C, loaded into the calorimeter, and equilibrated for 30 min. Lysozyme (3, 6, 50, and 100 mg/mL) was titrated into 0.01 μg/mL NS3/4A in 15 injections: one 1.5 μL, followed by 3 μL injections at 500 s intervals. This resulted in approximately 0.5, 1, 10, and 15 mg/mL lysozyme in the cell after the 11th injection, respectively. Measurements were performed at 37°C with stirring at 250 rpm and thermal power recorded every 1 s. The baseline was recorded for 200 s before the first and after the last injection. Corresponding lysozyme‐to‐buffer, buffer‐to‐NS3/4A, and buffer‐to‐buffer controls were performed. Experiment was repeated twice. Thermograms were baseline‐corrected using NanoAnalyze software (TA Instruments) and are shown as the mean heat rate (*n* = 2).

### 4.5 Intrinsic fluorescence measurements

NS3/4A (0 and 1 μg/mL final) was incubated in buffer or in 200 g/L PEGs and Ficoll 70, and 150 g/L Dextran 70 (the highest concentrations at which a consistent effect on NS3/4A activity was observed, Figure 2) in sealed black 96‐well F‐bottom plates (100 μL/well) at room temperature for 10 min, then centrifuged at room temperature for 10 min at 2000 rpm. Intrinsic fluorescence was recorded on an Enspire 2300 Multilabel Reader (PerkinElmer) with excitation at 280 nm. Emission spectra were collected from 300 to 400 nm every 3 nm during a temperature ramp 30–65°C at 1°C/min, with 5°C sampling intervals and an additional 1 min equilibration delay at each step. Instrument settings were: excitation/emission slit widths 5 nm, measurement height 7.5 mm, and photomultiplier tube voltage 750 V. Fluorescence was measured in two independent runs (*n* = 2) with three scans per run. The resulting spectra were averaged, baseline‐corrected, smoothed using the Savitzky–Golay method, and reported as mean in RFU. Samples without NS3/4A (0 μg/mL) served as background controls. The ratio of fluorescence intensities at 350 and 330 nm (350/330) and the fluorescence yields, quantified as AUC, were reported as mean ± SD. Two‐way ANOVA (independent variables: crowder type and temperature point) followed by Dunnett's post hoc test was used to evaluate the statistical significance of the 350/330 ratio relative to its value at 35°C. Two‐way ANOVA (independent variables: crowder type and NS3/4A presence) followed by Tukey's post hoc multiple‐comparison test was used to evaluate pairwise significant differences of AUC at each temperature point between all tested conditions. Multiplicity‐adjusted *p* values <0.05 were considered statistically significant. Intrinsic fluorescence intensity and AUC of NS3/4A in dilute conditions as a function of temperature were fitted to a Boltzmann sigmoidal model ($R^{2}>0.8$; Figure 4c and $R^{2}>0.9$; Figure S9B, respectively).

### 4.6 DCVJ fluorescence measurements

The experiment was performed similarly to the NS3/4A intrinsic fluorescence measurements, but with DCVJ (0 and 4 μM final) instead of protease. In short, DCVJ was dissolved in DMSO (400 μM stocks) and added to milli‐Q water, buffer, and buffer containing 200 g/L PEGs or Ficoll 70, or 150 g/L Dextran 70 in black 96‐well F‐bottom plates (100 μL/well). Plates were sealed, incubated and centrifuged as described above. Fluorescence spectra were recorded using the same plate reader and instrument settings, with excitation at 440 nm and emission collected from 460 to 650 nm every 3 nm. The same temperature‐ramp protocol described above was applied. Fluorescence was measured in three independent runs (*n* = 3), with three scans per run. The resulting spectra were averaged, baseline‐corrected, smoothed using the Savitzky–Golay method, and reported as mean in RFU. Samples without DCVJ (0 μM) served as background controls (Figure S10B). Fluorescence yields were quantified as AUC and reported as mean ± SEM. Two‐way ANOVA (independent variables: crowder type and DCVJ presence) followed by Tukey's post hoc multiple‐comparison test was used to evaluate pairwise significant differences in AUC at each temperature point between all tested conditions. Multiplicity‐adjusted *p* values <0.05 were considered statistically significant.

## AUTHOR CONTRIBUTIONS

**ML**: investigation, methodology, formal analysis, validation, writing—original draft, editing. **JT**: conceptualization, project administration, funding acquisition, data curation, resources, supervision, writing—review and editing.

## FUNDING INFORMATION

The funding for this study was provided by the National Science Centre, Poland (NCN). 2022/47/O/NZ1/01933 is the grant number.

## CONFLICT OF INTEREST STATEMENT

The authors have no competing interests to declare that are relevant to the content of this article.

## Supporting information


**Data S1.** Supporting Information.

## Data Availability

The data are available in Supporting Information S1 or RepOD repository at https://doi.org/10.18150/9ZPP7S.
