# Supplementary material for: Crowder‐specific modulation of hepatitis C virus NS3/4A protease activity and local structural dynamics
Source: Protein Sci. 2026 Oct 8;35(11):e70810. doi: 10.1002/pro.70810 (PMC13648321; doi:10.1002/pro.70810)
Supplement: Supplementary file 1 [file PRO-35-e70810-s001.pdf]

Supporting Materials for:

Crowder-specific modulation of hepatitis C virus NS3/4A protease activity and local structural dynamics

Małgorzata Lobka[1] and Joanna Trylska[1*]

[1]University of Warsaw, Centre of New Technologies, S. Banacha 2c, Warsaw, 02-097, Poland.

*Corresponding author(s). E-mail(s): joanna@cent.uw.edu.pl;
Contributing authors: m.lobka@cent.uw.edu.pl;

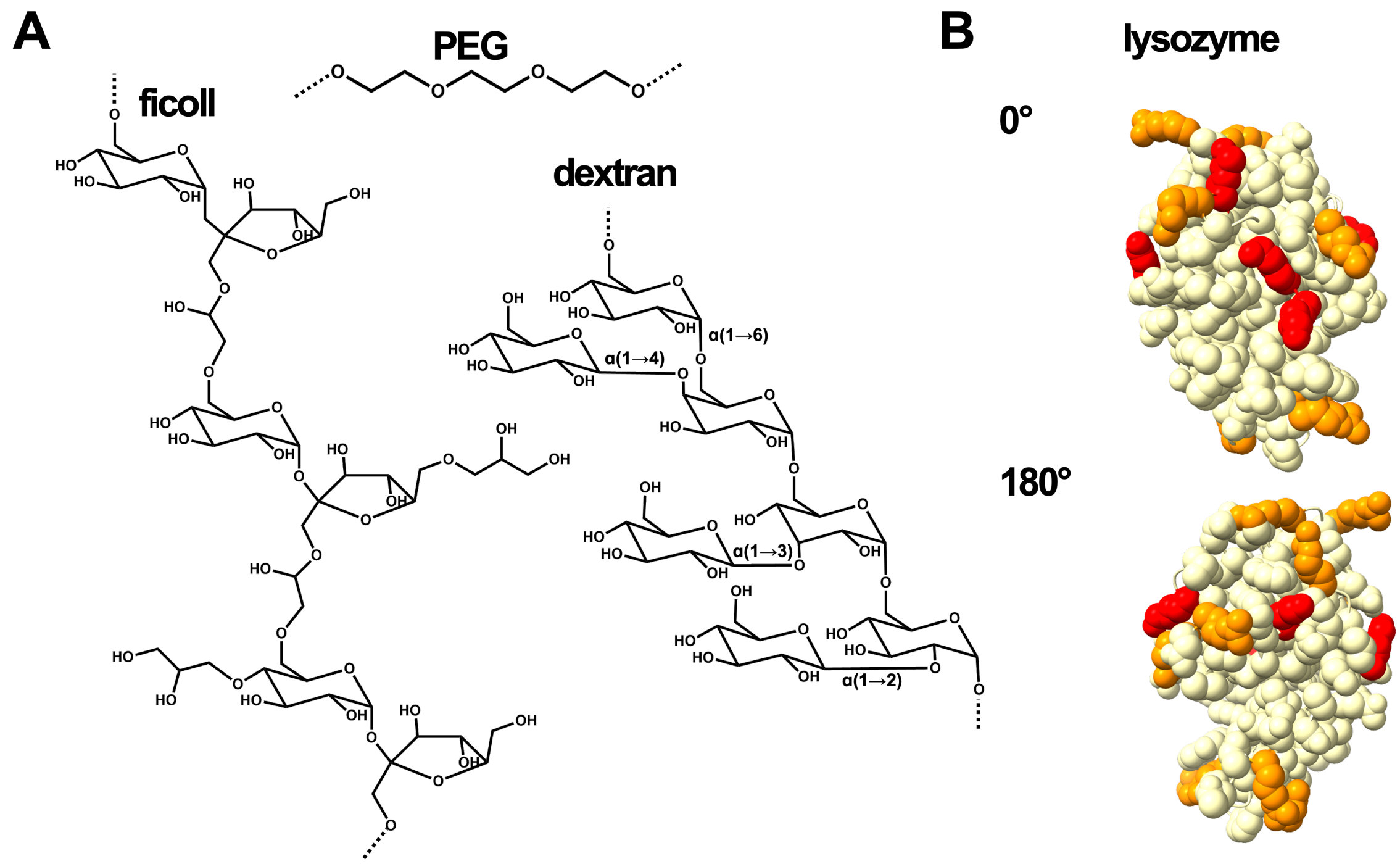

Fig. S1 Graphical representation of studied crowding agents. (A) Structural fragments of polyethylene glycol (PEG), ficoll, and dextran. Ficoll fragment is represented as a polysucrose chain randomly cross-linked with epichlorohydrin. Dextran fragment is represented as a linear backbone of α(1→6)-linked D-glucopyranosyl units with potential branches of D-glucose linked to the backbone by α(1→4), α(1→3), and α(1→2)-glycosidic bonds (the degree of branching varies by dextran class and type). (B) Hen egg-white lysozyme unit visualized with ChimeraX [1] as van der Waals spheres, with highlighted surface Lys (red) and Arg (orange) residues, based on crystal structure PDB ID: 1LYZ [2].

Table S1 Side-by-side kinetic parameter comparison of NS3/4A activity under different thawing methods in dilute conditions. Parameters were derived from Michaelis-Menten fits (**Figure S2**) and are reported as mean ± SEM, (n = 3). Previous protocol denotes direct thawing at room temperature (data are taken from [3]), while revised protocol denotes preincubation on ice before room-temperature equilibration (data determined in this study).

thawing method	v_{max} [µM/s]	K_{M} [µM]	k_{cat} [s^{-1}]	$k_{\mathrm{cat}}/K_{\mathrm{M}}$ [µM^{-1}s^{-1}]
room-temperature	3.4 ± 0.1	7 ± 1	1700 ± 100	240 ± 40
on ice	7.8 ± 0.1	19 ± 1	$3,900 \pm 100$	205 ± 5

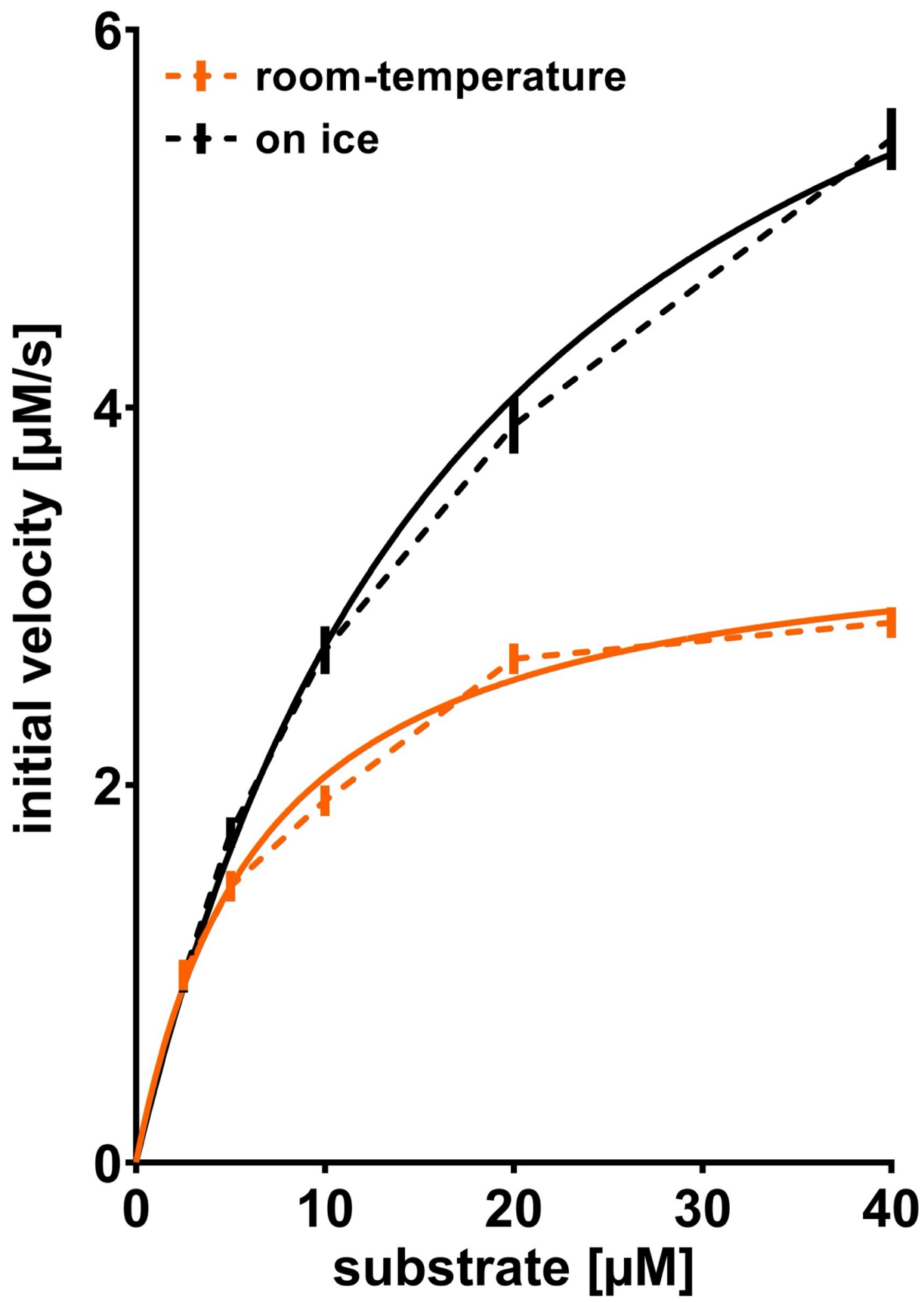

Fig. S2 Side-by-side kinetic comparison of NS3/4A activity under different thawing methods in dilute conditions. Initial velocities were determined using 0-40 μM substrate and 0.05 μg/mL enzyme. Data represent mean ± SEM (n = 3) and were fitted to the Michaelis-Menten equation across the entire substrate range. Previous and revised protocols defined as in **Table S1**.

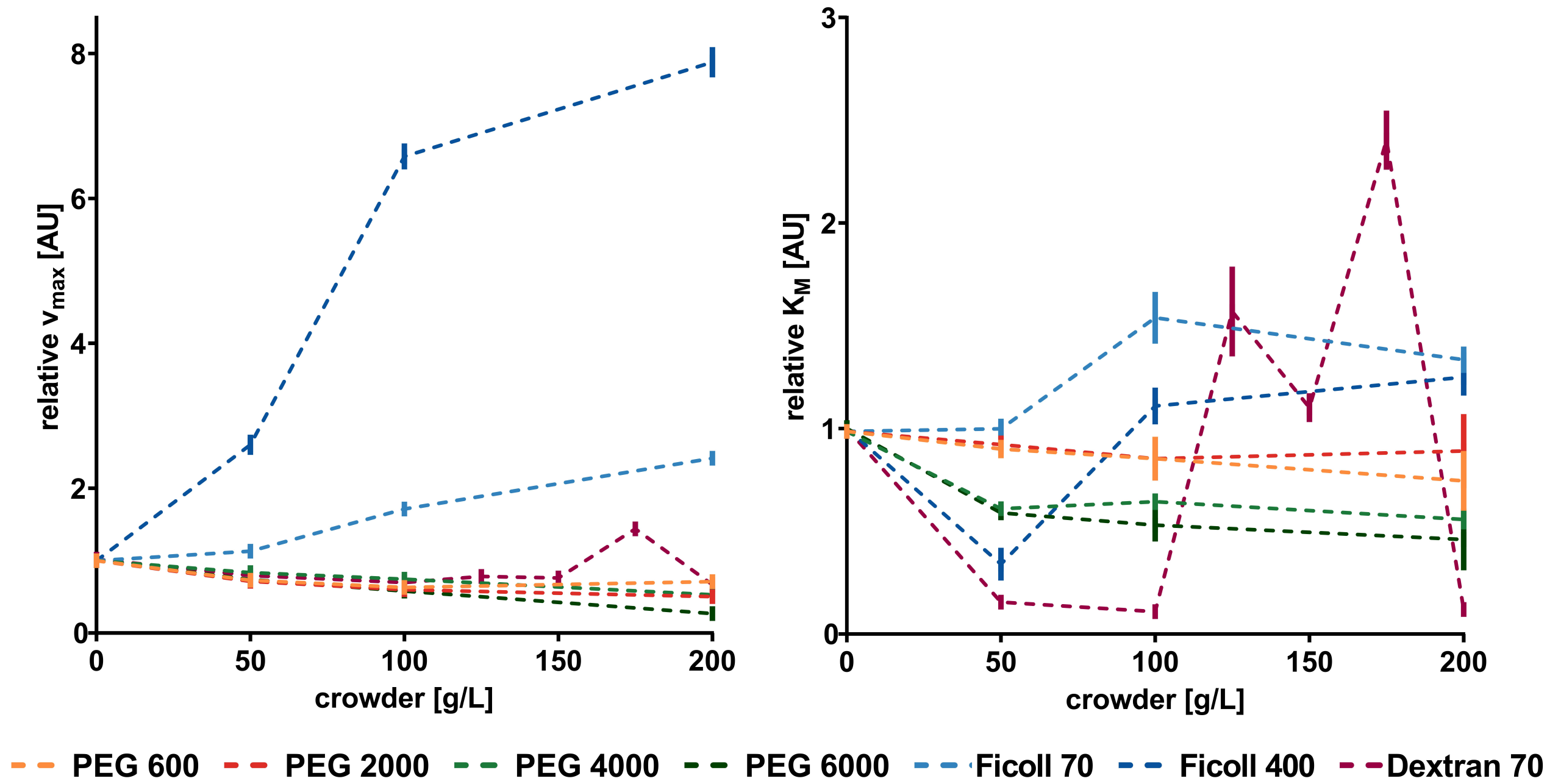

Fig. S3 Comparison of relative kinetic parameters of the NS3/4A-mediated hydrolysis under crowded conditions. Maximal reaction rate ($v_{\max}$) and Michaelis constant (K_M) were determined from fitting initial velocities to the Michaelis-Menten equation at the 0–40 µM substrate range (**Figure 2A** in the main text) and are shown as mean ± SEM (n = 3, each in duplicate) relative to control dilute conditions. Data for PEG 2000, PEG 4000, Ficoll 70, and Dextran 70 were experimentally determined in this study. Data for PEG 600, PEG 6000, and Ficoll 400 were taken from [3]. The effect at 175 g/L Dextran 70 deviated from observed trend, and we were not able to identify the definite cause. We therefore report these data (see: **Figure 2** and **Table 1**) for completeness but refrain from mechanistic interpretation.

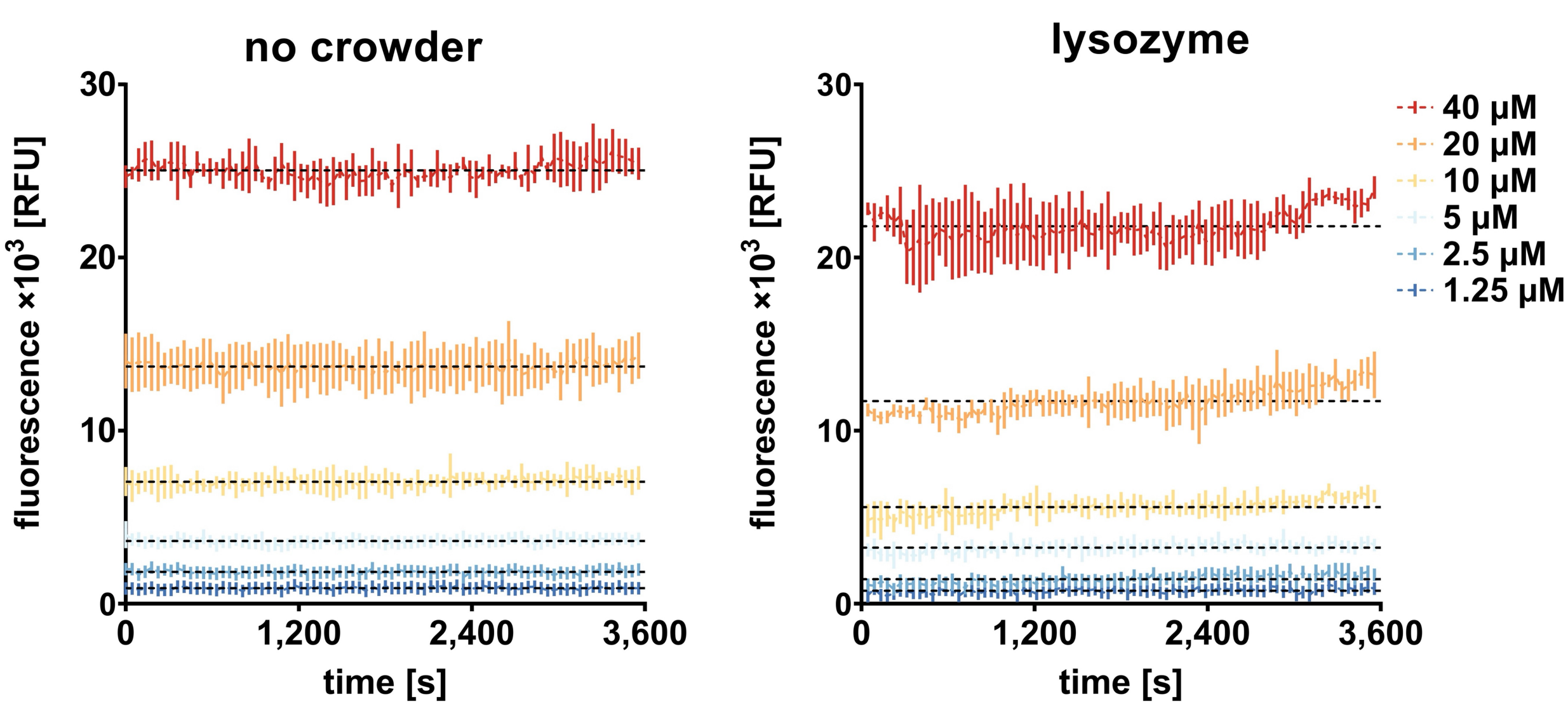

Fig. S4 FRET-substrate fluorescence stability under dilute conditions and in the presence of lysozyme. Fluorescence signal from 0–40 µM FRET-substrate was recorded in the absence of NS3/4A protease under dilute conditions (no crowder) and in the presence of 75 g/L lysozyme to confirm the absence of substrate cleavage. Data were baseline-corrected and are presented as mean ± SD (n = 2). Black dashed lines represent the average fluorescence across the entire measurement period, indicating no visible increase in signal during the initial phase.

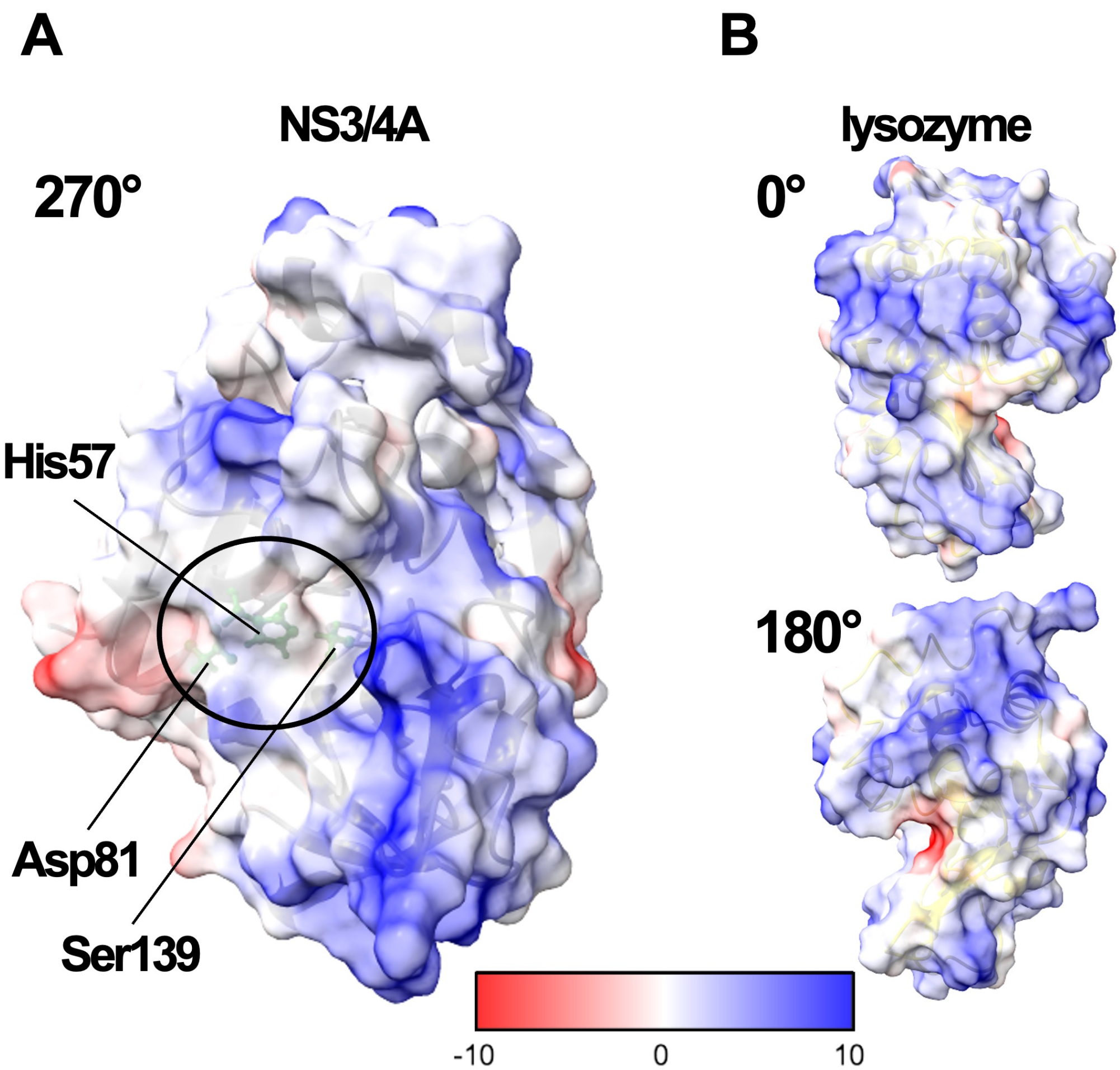

Fig. S5 Electrostatic surface potential of NS3/4A and lysozyme. Electrostatic surface distribution of (A) NS3/4A protease (grey ribbon) and (B) lysozyme (yellow ribbon), visualized using ChimeraX [1]. The color of the semitransparent surface maps positive (blue) and negative electrostatic potential (red); coulombic electrostatic potential values are given in arbitrary units. Residues forming NS3/4A catalytic triad are marked in green, and a black circle marks active site.

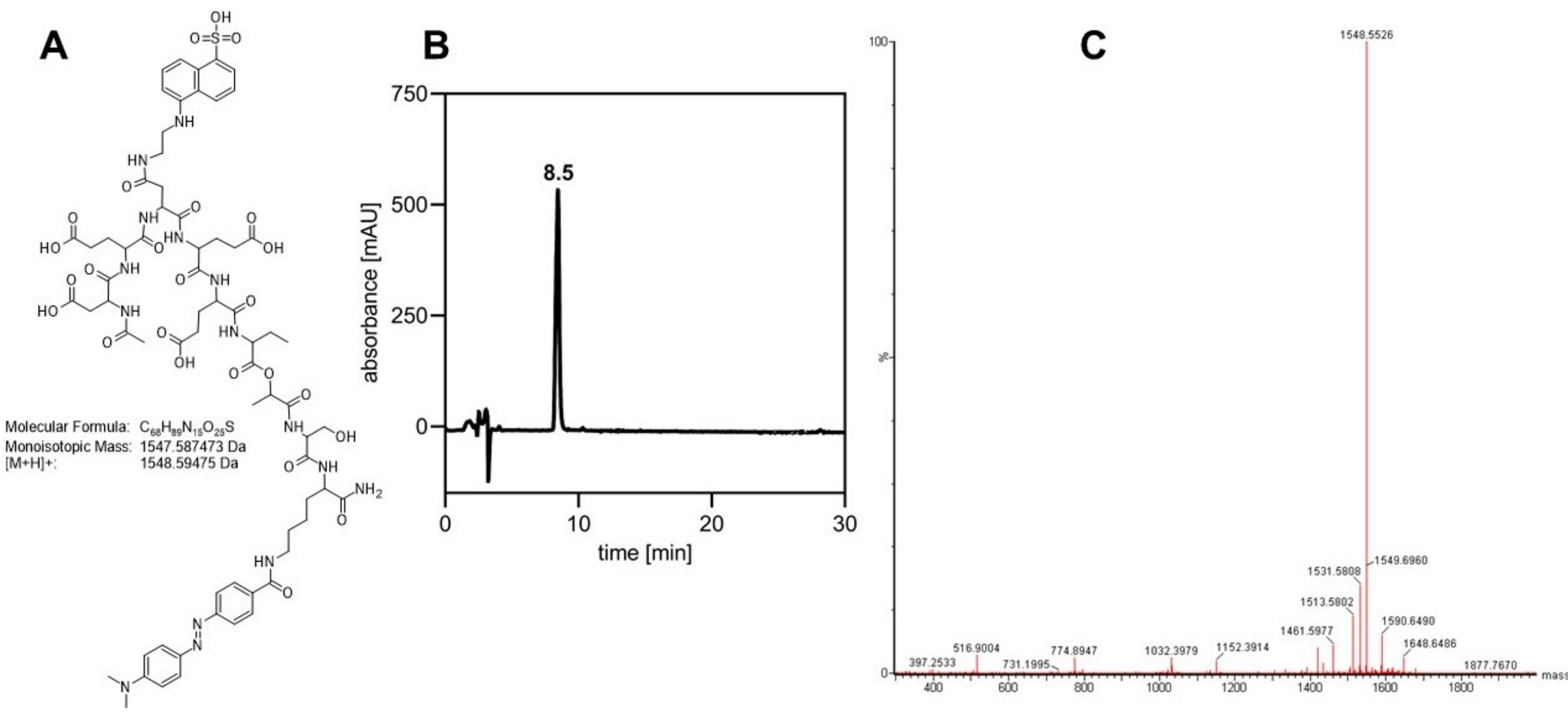

Fig. S6 The structure, chromatogram and mass spectrum of the FRET-substrate. (A) Chemical structure of Ac-DED(EDANS)EEAbu-ψ-[COO]-ASK(DABCYL)-NH_2 with calculated monoisotopic and quasi-molecular ion masses. (B) Analytical RP-HPLC chromatogram. The substrate eluted at 8.5 min. Substrate purity was determined to be $> 95\%$ by HPLC. (C) ESI-MS spectrum. The observed $m/z = 1548.6$ matched the calculated value for $[\mathrm{M+H}]^+$ confirming the product's identity.

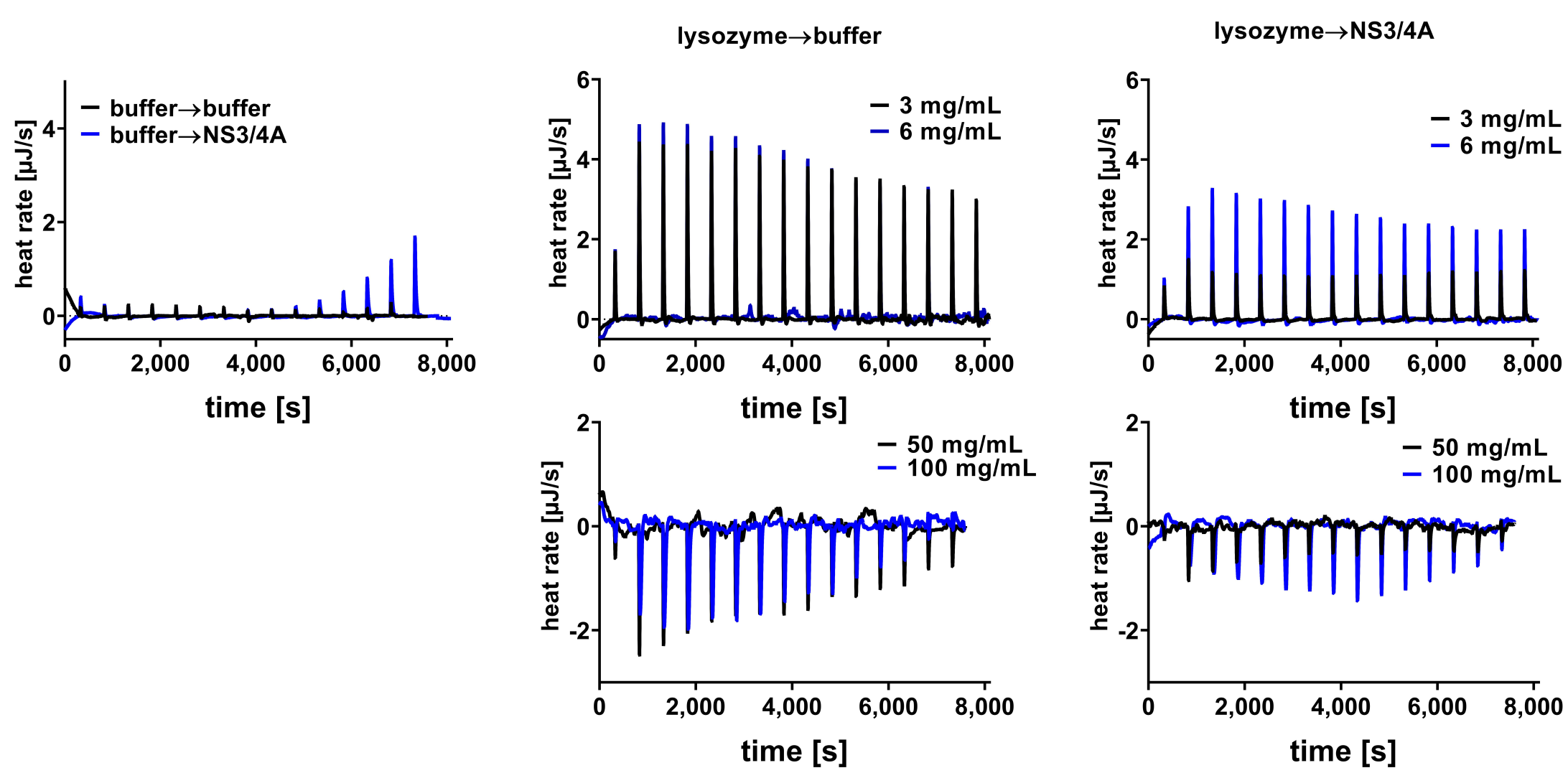

Fig. S7 ITC thermograms of lysozyme and NS3/4A under different titration conditions. Buffer was titrated into buffer or NS3/4A as controls. Lysozyme (3, 6, 50, and 100 mg/mL) was titrated into buffer or NS3/4A. Measurements were performed at 37°C with 0.01 µg/mL NS3/4A in the cell. The first injection was 1.5 µL, followed by 3 µL injections at 500 s intervals. Data are shown as baseline-corrected mean heat rates from two independent experiments (n = 2). The low enzyme concentration limited the expected signal from interactions, while heat of lysozyme dilution prevented reliable correction of the lysozyme-to-NS3/4A signal and subsequent fitting to standard binding models.

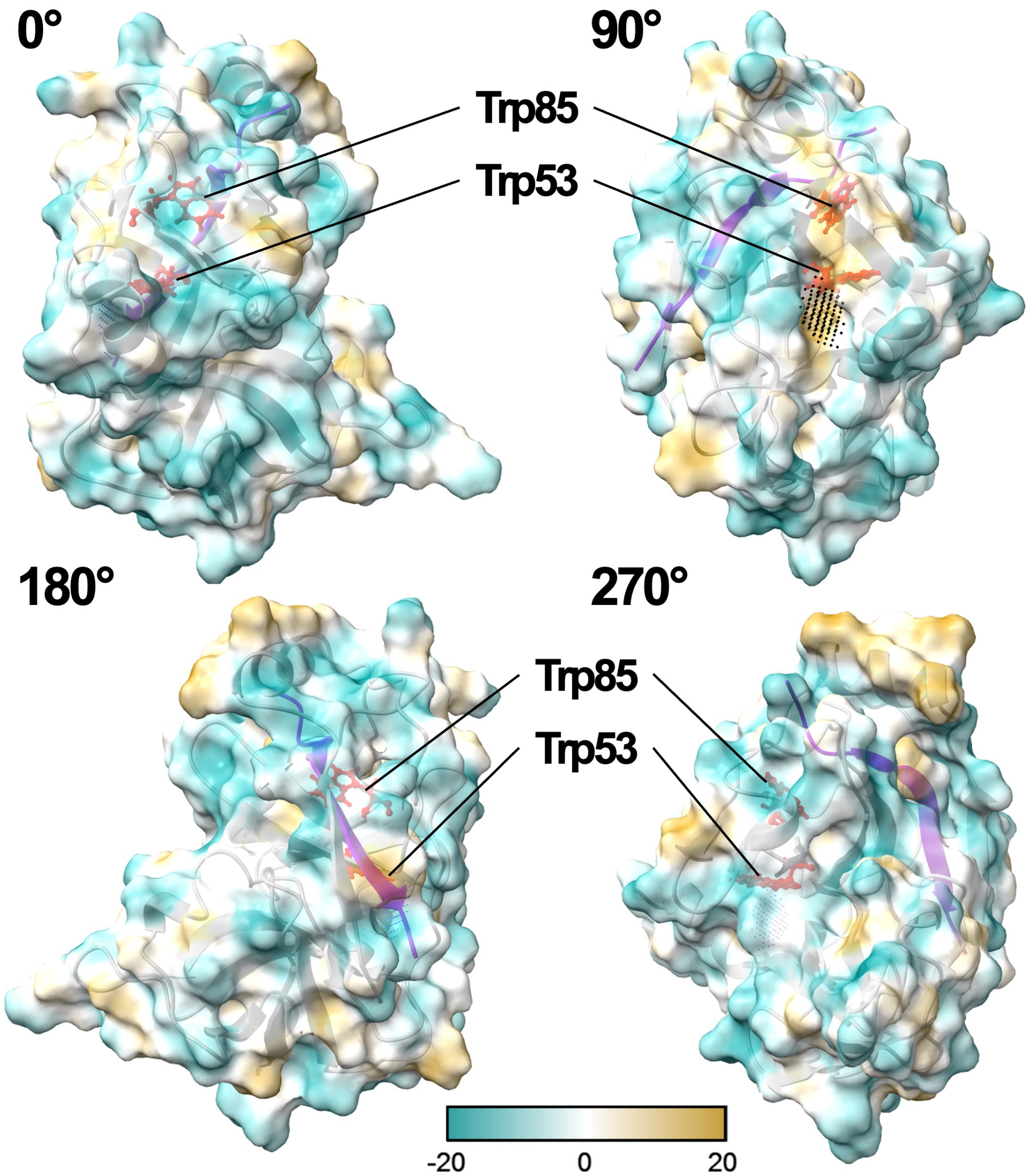

Fig. S8 Hydrophobic surface distribution of the NS3/4A protease. Rotational views of the NS3 protease (grey ribbon) in complex with the NS4A cofactor (violet ribbon), visualized using ChimeraX [1]. The color of the semitransparent surface maps local hydrophobicity (gold) and hydrophilicity (cyan); molecular hydrophobicity potential values are given in arbitrary units. Tryptophans are marked in red. The solvent-exposed surface area is 4.1 Å² for Trp53, and around 1 Å² for Trp85. The hydrophobic cavity of approximately 35 Å² lined by Trp53 was identified using pyKVFinder [4] and is indicated by black dots. The inner probe radius of 1.4 Å corresponded to the approximate size of a water molecule.

Table S2 Contacts of Trp53 in the NS3/4A protease. Contacts were analyzed in ChimeraX [1] using the NS3/4A model based on [3] and crystal structure 4JMY [5]. Atoms were counted as contacting when located within 0.4 Å of van der Waals contact (overlap). Positive and near-zero overlap values indicate tight packing that contributes to Trp shielding. Intra-residue contacts were excluded. The chain identifier is noted as /A for the NS3 protease. Atom numbering is shown as in the PDB 4JMY structure [5].

atom1	atom2	overlap [Å]	distance [Å]
/A TRP 53 HN	/A THR 46 O	0.218	1.862
/A TRP 53 O	/A ALA 45 HA	0.211	2.269
/A TRP 53 O	/A ALA 45 CA	0.092	3.088
/A TRP 53 O	/A THR 46 HN	0.091	1.989
/A TRP 53 CE2	/A VAL 51 HG12	0.039	2.661
/A TRP 53 CH2	/A TYR 75 CD2	0.038	3.362
/A TRP 53 NE1	/A VAL 51 HB	0.023	2.602
/A TRP 53 CE2	/A VAL 51 CG1	0.019	3.381
/A TRP 53 HA	/A VAL 83 O	0.002	2.478
/A TRP 53 CE3	/A LEU 82 HD11	-0.035	2.735
/A TRP 53 HZ2	/A MET 179 SD	-0.049	2.831
/A TRP 53 NE1	/A VAL 51 CG1	-0.053	3.378
/A TRP 53 HE3	/A LEU 82 CD1	-0.059	2.759
/A TRP 53 CZ2	/A MET 175 HB1	-0.077	2.777
/A TRP 53 CE3	/A LEU 82 CD1	-0.098	3.498
/A TRP 53 CZ2	/A VAL 51 HG12	-0.109	2.809
/A TRP 53 NE1	/A VAL 51 CB	-0.114	3.439
/A TRP 53 HE3	/A VAL 83 O	-0.132	2.612
/A TRP 53 N	/A THR 46 O	-0.157	2.862
/A TRP 53 O	/A THR 46 N	-0.174	2.879
/A TRP 53 HE3	/A LEU 82 HD11	-0.189	2.189
/A TRP 53 CA	/A VAL 83 O	-0.223	3.403
/A TRP 53 CE3	/A LEU 82 CG	-0.223	3.623
/A TRP 53 HE1	/A VAL 51 HB	-0.234	2.234
/A TRP 53 O	/A ALA 45 CB	-0.242	3.422
/A TRP 53 HN	/A THR 46 C	-0.244	2.944
/A TRP 53 CE2	/A MET 175 HB1	-0.255	2.955
/A TRP 53 CE3	/A VAL 83 O	-0.265	3.445
/A TRP 53 O	/A ALA 45 C	-0.265	3.445
/A TRP 53 CD1	/A VAL 48 HB	-0.277	2.977
/A TRP 53 CD1	/A MET 175 SD	-0.280	3.762
/A TRP 53 HH2	/A MET 179 HG2	-0.290	2.290
/A TRP 53 CD1	/A VAL 51 O	-0.292	3.472
/A TRP 53 CZ3	/A LEU 82 HG	-0.295	2.995
/A TRP 53 CH2	/A TYR 75 CG	-0.300	3.700
/A TRP 53 CE3	/A LEU 82 HG	-0.301	3.001
/A TRP 53 CZ3	/A TYR 75 HB2	-0.317	3.017
/A TRP 53 HH2	/A TYR 75 CD2	-0.323	3.023
/A TRP 53 CD1	/A VAL 51 CG1	-0.331	3.731
/A TRP 53 CD2	/A VAL 51 CG1	-0.345	3.745
/A TRP 53 HE3	/A LEU 82 CG	-0.347	3.047
/A TRP 53 CZ2	/A MET 179 SD	-0.353	3.835
/A TRP 53 O	/A THR 54 HB	-0.360	2.840
/A TRP 53 HA	/A GLY 84 HA2	-0.363	2.363
/A TRP 53 CZ3	/A LEU 82 CG	-0.374	3.774
/A TRP 53 HH2	/A TYR 75 CG	-0.378	3.078
/A TRP 53 CG	/A MET 175 SD	-0.390	3.872
/A TRP 53 HD1	/A VAL 48 HB	-0.393	2.393
/A TRP 53 CE3	/A VAL 83 C	-0.394	3.794
/A TRP 53 CD1	/A VAL 51 HG11	-0.395	3.095
/A TRP 53 CZ2	/A VAL 51 CG1	-0.397	3.797

Table S3 Contacts of Trp85 in the NS3/4A protease. Contacts were analyzed as in **Table S2**. The chain identifiers are noted as /A for the NS3 protease and /D for the NS4A cofactor.

atom1	atom2	overlap [Å]	distance [Å]
/A TRP 85 O	/A CYS 52 HN	0.261	1.819
/A TRP 85 CE2	/A LEU 64 HB2	0.101	2.599
/A TRP 85 NE1	/A GLN 73 CG	0.072	3.253
/A TRP 85 HN	/A CYS 52 O	0.024	2.056
/A TRP 85 CD2	/A LEU 64 HB2	-0.009	2.709
/A TRP 85 CD1	/A ILE 71 O	-0.010	3.190
/A TRP 85 CZ3	/A THR 54 CG2	-0.023	3.423
/A TRP 85 HD1	/A THR 72 C	-0.038	2.738
/A TRP 85 O	/A VAL 51 HG13	-0.054	2.534
/A TRP 85 O	/A CYS 52 N	-0.068	2.773
/A TRP 85 HD1	/A ILE 71 O	-0.073	2.553
/A TRP 85 NE1	/A GLN 73 HG2	-0.079	2.704
/A TRP 85 HE1	/A LYS 62 O	-0.083	2.163
/A TRP 85 NE1	/A LEU 64 N	-0.105	3.355
/A TRP 85 O	/A VAL 51 HA	-0.124	2.604
/A TRP 85 CD1	/A GLN 73 HA	-0.137	2.837
/A TRP 85 HA	/A PRO 86 HD1	-0.155	2.155
/A TRP 85 HH2	/A ALA 59 CB	-0.165	2.865
/A TRP 85 HZ3	/A THR 54 CG2	-0.176	2.876
/A TRP 85 CD1	/A GLN 73 N	-0.177	3.502
/A TRP 85 CZ2	/D VAL 225 HG21	-0.177	2.877
/A TRP 85 CH2	/A ALA 59 CB	-0.181	3.581
/A TRP 85 CD1	/A GLN 73 CA	-0.191	3.591
/A TRP 85 HD1	/A GLN 73 N	-0.191	2.816
/A TRP 85 CH2	/A THR 54 CG2	-0.201	3.601
/A TRP 85 CH2	/A THR 54 HG22	-0.206	2.906
/A TRP 85 O	/A VAL 51 CG1	-0.209	3.389
/A TRP 85 CZ3	/32 TIP 21098 H1	-0.213	2.913
/A TRP 85 HH2	/A ALA 59 HB3	-0.213	2.213
/A TRP 85 HB1	/A ILE 71 CG2	-0.218	2.918
/A TRP 85 NE1	/A GLN 73 HG1	-0.218	2.843
/A TRP 85 CZ3	/A THR 54 HG22	-0.225	2.925
/A TRP 85 HZ3	/32 TIP 21098 H1	-0.228	2.228
/A TRP 85 CZ3	/A THR 54 HG23	-0.228	2.928
/A TRP 85 HD1	/A THR 72 O	-0.232	2.712
/A TRP 85 O	/A VAL 51 CA	-0.235	3.415
/A TRP 85 CE2	/A LEU 64 CB	-0.237	3.637
/A TRP 85 NE1	/A LEU 64 HB2	-0.254	2.879
/A TRP 85 HZ2	/D VAL 225 HG21	-0.255	2.255
/A TRP 85 CD1	/A GLN 73 CG	-0.273	3.673
/A TRP 85 CD2	/A LEU 64 CB	-0.281	3.681
/A TRP 85 HZ2	/A LYS 62 O	-0.283	2.763
/A TRP 85 CZ2	/A ALA 59 HB1	-0.295	2.995
/A TRP 85 N	/A CYS 52 O	-0.306	3.011
/A TRP 85 CE2	/A GLN 73 HG2	-0.310	3.010
/A TRP 85 CD1	/A THR 72 C	-0.310	3.710
/A TRP 85 CB	/A PRO 86 HD1	-0.315	3.015
/A TRP 85 NE1	/A THR 63 C	-0.320	3.645
/A TRP 85 HE3	/A CYS 52 O	-0.321	2.801
/A TRP 85 HE1	/A GLN 73 CG	-0.332	3.032
/A TRP 85 CD1	/A LEU 64 CB	-0.334	3.734
/A TRP 85 NE1	/A LEU 64 CB	-0.336	3.661
/A TRP 85 HE1	/A GLN 73 HG1	-0.336	2.336
/A TRP 85 C	/A CYS 52 HN	-0.340	3.040
/A TRP 85 CZ2	/A LYS 62 O	-0.341	3.521
/A TRP 85 CE3	/A LEU 64 HD22	-0.346	3.046
/A TRP 85 O	/A VAL 51 C	-0.347	3.527
/A TRP 85 NE1	/A LYS 62 O	-0.348	3.053
/A TRP 85 CD1	/A GLN 73 HG2	-0.352	3.052
/A TRP 85 CZ2	/D VAL 225 CG2	-0.352	3.752
/A TRP 85 CH2	/D VAL 225 HG21	-0.355	3.055
/A TRP 85 HH2	/A THR 54 HG22	-0.357	2.357
/A TRP 85 C	/A VAL 51 HG13	-0.360	3.060
/A TRP 85 CG	/A LEU 64 CB	-0.362	3.762
/A TRP 85 HB1	/A ILE 71 HG21	-0.371	2.371
/A TRP 85 CH2	/A ALA 59 HB1	-0.382	3.082
/A TRP 85 CG	/A LEU 64 HB2	-0.383	3.083
/A TRP 85 HZ2	/D VAL 225 CG2	-0.388	3.088
/A TRP 85 HZ3	/A THR 54 HG22	-0.391	2.391

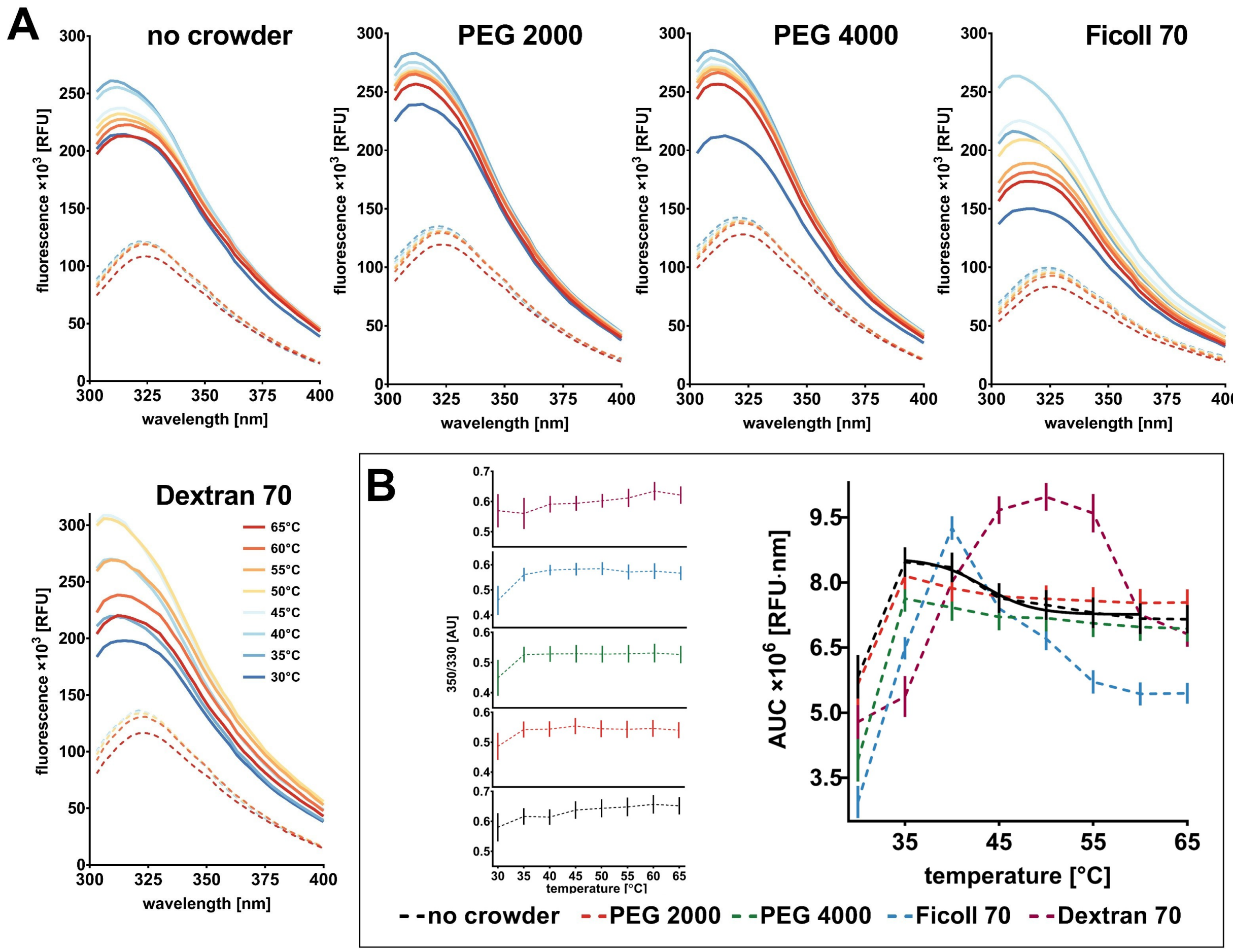

Fig. S9 Intrinsic fluorescence of the NS3/4A enzyme under dilute and crowded conditions. (A) Fluorescence emission spectra were recorded in control dilute conditions and in 200 g/L PEGs and Ficoll 70, and in 150 g/L Dextran 70. Spectra for protein-containing samples (solid lines) and background control (dashed lines) were averaged from three scans and smoothed with the Savitzky-Golay method. (B) Baseline-corrected fluorescence 350/330 ratio and yield quantified as area under the emission curve (AUC; mean $\pm$ SD, $n = 2$, three scans each) as functions of temperature. For dilute conditions, data were fitted to a Boltzmann sigmoidal curve across 35-60°C, with the midpoint estimated at around 43°C.

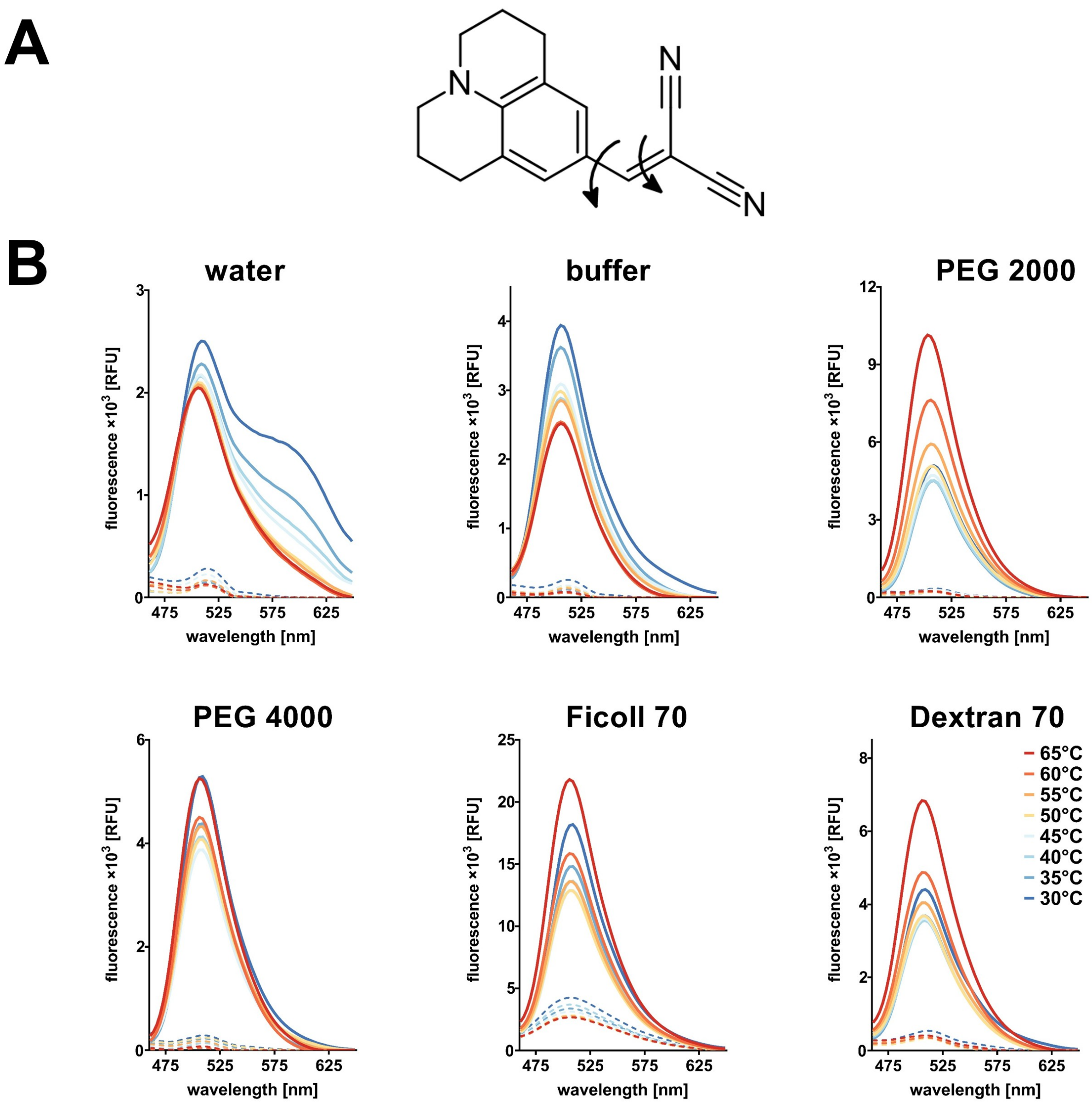

Fig. S10 DCVJ structure and its fluorescence under dilute and crowded conditions at different temperature points. (A) 9-(2,2-dicyanovinyl)julolidine (DCVJ) chemical structure. Intramolecular rotation relaxation paths are marked with black arrows [6]. (B) DCVJ fluorescence emission spectra were recorded in water, control dilute conditions (buffer), and in 200 g/L PEGs and Ficoll 70, and in 150 g/L Dextran 70. Spectra for DCVJ-containing samples (solid lines) and background control (dashed lines) were averaged from three scans and smoothed with the Savitzky-Golay method.

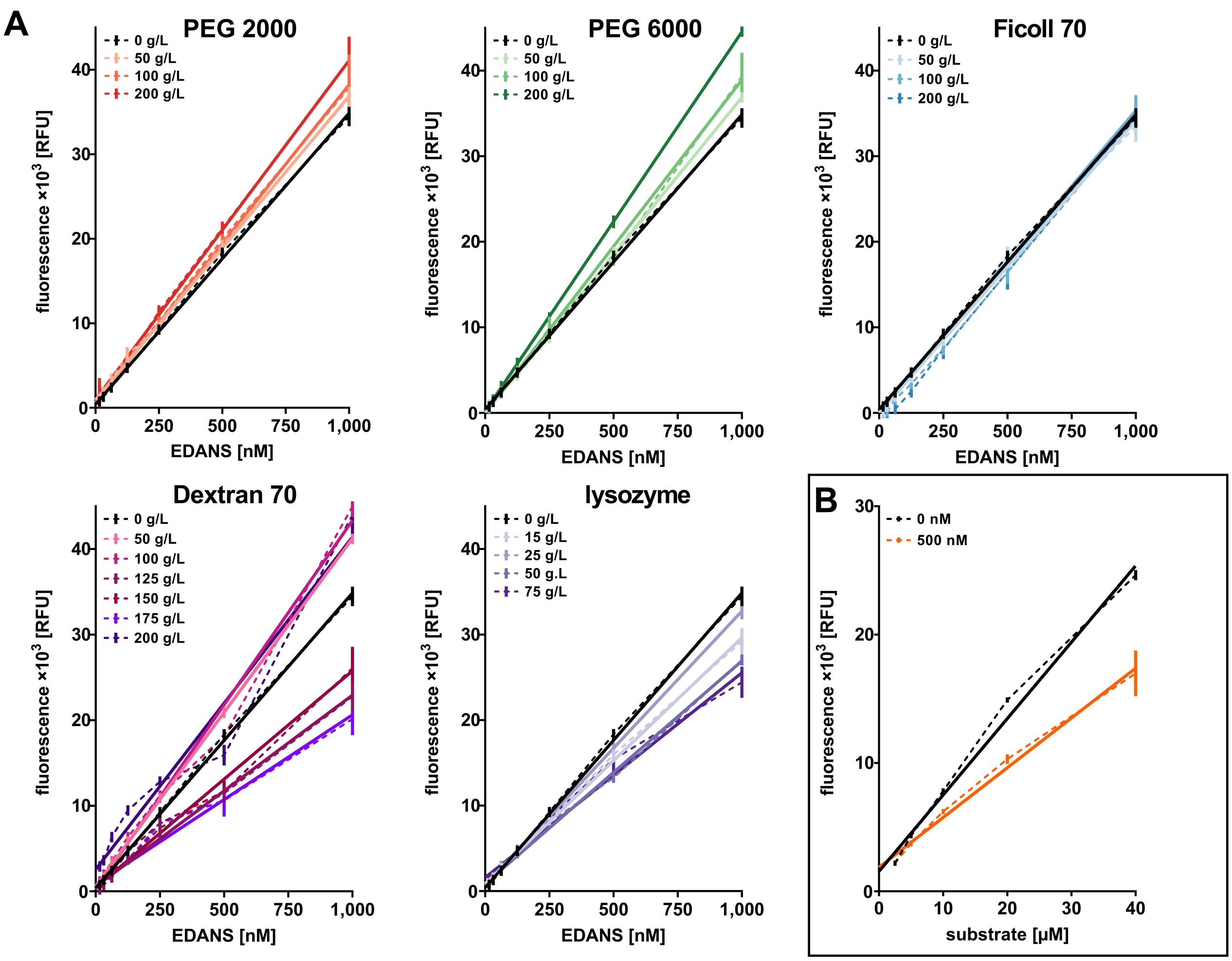

Fig. S11 EDANS calibration under dilute and crowded conditions. (A) Fluorescence response was tested with excitation at 340 nm and emission at 490 nm for 0-1,000 nM EDANS under dilute conditions and in 50-200 g/L polymer crowders, and 15-75 g/L lysozyme. Data points represent the mean ± SEM (n = 3, each in duplicate) and were fitted to a linear regression model (solid lines). (B) Inner filter effects were evaluated in assay buffer for 0-40 µM substrate in the absence and presence of 500 nM EDANS. Data points represent the mean ± SD (n = 2, each in duplicate) and were fitted to a linear regression model (solid lines).

References

[1] Meng, E.C., Goddard, T.D., Pettersen, E.F., Couch, G.S., Pearson, Z.J., Morris, J.H., Ferrin, T.E.: UCSF ChimeraX: Tools for structure building and analysis. Protein Science **32**(11), 4792 (2023) https://doi.org/10.1002/pro.4792

[2] Diamond, R.: Real-space refinement of the structure of hen egg-white lysozyme (1974). https://doi.org/10.1016/0022-2836(74)90598-1

[3] Popielec, A., Ostrowska, N., Wojciechowska, M., Feig, M., Trylska, J.: Crowded environment affects the activity and inhibition of the NS3/4A protease. Biochimie **176**, 169–180 (2020) https://doi.org/10.1016/j.biochi.2020.07.009

[4] Guerra, J.V.D.S., Ribeiro-Filho, H.V., Jara, G.E., Bortot, L.O., Pereira, J.G.C., Lopes-de-Oliveira, P.S.: pyKVFinder: An efficient and integrable python package for biomolecular cavity detection and characterization in data science. BMC Bioinformatics **22**(1), 607 (2021) https://doi.org/10.1186/s12859-021-04519-4

[5] Lemke, C.T.: Crystal structure of HCV NS3/NS4A protease complexed with DDIVPC peptide (2013). https://doi.org/10.2210/pdb4jmy/pdb

[6] Yang, S., Han, K.: Effects of solvent dielectric constant and viscosity on two rotational relaxation paths of excited 9-(dicyanovinyl) julolidine. The Journal of Physical Chemistry A **120**(27), 4961–4965 (2016) https://doi.org/10.1021/acs.jpca.5b12612
